# Smoke-free legislation and the incidence of paediatric respiratory infections and wheezing/asthma: interrupted time series analyses in the four UK nations

**DOI:** 10.1038/srep15246

**Published:** 2015-10-14

**Authors:** Jasper V. Been, Lisa Szatkowski, Tjeerd-Pieter van Staa, Hubert G. Leufkens, Onno C. van Schayck, Aziz Sheikh, Frank de Vries, Patrick Souverein

**Affiliations:** 1 Division of Neonatology, Erasmus University Medical Centre – Sophia Children’s Hospital, Rotterdam, Netherlands; 2 Centre of Medical Informatics, Usher Institute of Population Health Sciences and Informatics, The University of Edinburgh, Edinburgh, United Kingdom; 3 Department of Paediatrics, Maastricht University Medical Centre, Maastricht, Netherlands; 4 School for Public Health and Primary Care (CAPHRI), Maastricht University, Maastricht, Netherlands; 5 UK Centre for Tobacco and Alcohol Studies, University of Nottingham, Division of Epidemiology and Public Health, Nottingham City Hospital, Nottingham, United Kingdom; 6 London School of Hygiene and Tropical Medicine, London, United Kingdom; 7 Division of Pharmacoepidemiology and Clinical Pharmacology, Utrecht Institute of Pharmaceutical Sciences, Utrecht University, Utrecht, Netherlands; 8 Division of General Internal Medicine and Primary Care, Brigham and Women’s Hospital/Harvard Medical School, Boston, USA; 9 Department of Clinical Pharmacy & Toxicology, Maastricht University Medical Centre, Maastricht, the Netherlands; 10 MRC Epidemiology Lifecourse Unit, Southampton General Hospital, Southampton, United Kingdom

## Abstract

We investigated the association between introduction of smoke-free legislation in the UK (March 2006 for Scotland, April 2007 for Wales and Northern Ireland, and July 2007 for England) and the incidence of respiratory diseases among children. We extracted monthly counts of new diagnoses of wheezing/asthma and RTIs among children aged 0–12 years from all general practices in the Clinical Practice Research Datalink during 1997–2012. Interrupted time series analyses were performed using generalised additive mixed models, adjusting for underlying incidence trends, population size changes, seasonal factors, and pandemic influenza, as appropriate. 366,642 new wheezing/asthma diagnoses and 4,324,789 RTIs were observed over 9,536,003 patient-years. There was no statistically significant change in the incidence of wheezing/asthma after introduction of smoke-free legislation in England (incidence rate ratio (IRR) 0.94, 95% CI 0.81–1.09) or any other UK country (Scotland: IRR 0.99, 95% CI 0.83–1.19; Wales: IRR 1.09, 95% CI 0.89–1.35; Northern Ireland: IRR 0.96, 95% CI 0.76–1.22). Similarly no statistically significant changes in RTI incidence were demonstrated (England: IRR 0.95, 95% CI 0.86–1.06; Scotland: IRR 0.96, 95% CI 0.83–1.11; Wales: IRR 0.97, 95% CI 0.86–1.09; Northern Ireland: IRR 0.90, 95% CI 0.79–1.03). There were no demonstrable reductions in the incidence of paediatric wheezing/asthma or RTIs following introduction of smoke-free legislation in the UK.

Exposure to second-hand smoke (SHS) is estimated to be responsible for over 600,000 deaths and almost 11 million disability-adjusted life years (DALYs) worldwide each year^1^. Children account for over a quarter of these deaths and over half of the associated DALYs, which are almost entirely attributable to respiratory diseases^1^. There is clear evidence demonstrating that SHS exposure in the population can effectively be reduced by the implementation of legislation prohibiting smoking in enclosed public places and the workplace^2^. Associations between the introduction of smoke-free legislation and reductions in respiratory, cardiovascular, and cerebrovascular hospitalisations among adults are well recognised, and these are most pronounced in countries with the most comprehensive laws^3^. However, comprehensive smoke-free laws currently cover only about a sixth of the world’s population^4^.

Associations between smoke-free legislation and improved early-life outcomes are now increasingly becoming established. In a recent meta-analysis, 10% reductions in the incidence of preterm birth and of paediatric asthma exacerbations requiring hospital attendance were demonstrated following introduction of smoke-free laws^5^. Following the introduction of smoke-free legislation in England, a reduction in hospital admissions for acute RTIs among children was furthermore identified^6^. These observations are in line with the large body of epidemiological and mechanistic literature demonstrating that SHS exposure is associated with a range of paediatric respiratory diseases, including RTIs and asthma^7^,^8^,^9^. Reduced forced expiratory volume in one second (FEV1), increased airway hyper-reactivity and allergic sensitisation, and impairment of mucociliary clearance and the host response to infections have all been implicated as likely mediators of these associations^10^,^11^,^12^.

As the majority of the disease burden of paediatric respiratory diseases−including asthma and RTIs−lies in primary care, the lack of studies assessing the impact of smoke-free legislation on GP consultations is an important knowledge gap in the appreciation of the association between smoke-free legislation and child health^5^.

We aimed to study the association between the introduction of smoke-free legislation in each of the four countries in the UK and incidence changes in asthma and RTIs in general practices among children aged 12 and younger.

## Methods

This study was performed according to a pre-specified protocol that was approved by the Independent Scientific Advisory Committee of the Clinical Practice Research Datalink (CPRD; reference: 13_156 (see Supplementary Methods online)). We investigated the impact of smoke-free legislation on the incidence of RTIs and new diagnoses of wheezing/asthma among children aged ≤12 years who contributed data to CPRD during 1997-2012. Separate analyses were undertaken for each UK country. This was important because smoke-free legislation was introduced at different time points.

### Ethical approval

The CPRD has been granted Multiple Research Ethics Committee approval (05/MRE04/87) to undertake purely observational studies. The work of CPRD is also covered by National Information Governance Board Ethics and Confidentiality Committee approval ECC 5-05 (a) 2012. This study was furthermore reviewed by the National Health Services South East Scotland Research Ethics Service and The University of Edinburgh’s Centre for Population Health Sciences Ethics Review Group, who provided exemptions from formal ethical assessment based on the use of anonymised, unidentifiable data.

### Introduction of the smoke-free laws

The intervention under study was the law prohibiting smoking in enclosed public places and the workplace that was implemented at different time points in each country: 26 March 2006 in Scotland^13^, 2 April 2007 in Wales^14^, 30 April 2007 in Northern Ireland^15^, and 1 July 2007 in England^16^. From these dates onwards, smoking was prohibited in virtually all enclosed public places and workplaces; compliance with the smoke-free law has been high in each country at 97 to 98%^13^,^14^,^17^,^18^.

### Outcomes

We stipulated two primary outcomes *a priori*: the monthly incidence of new diagnoses of wheezing/asthma and the monthly incidence of RTI episodes. Wheezing/asthma was considered newly diagnosed when a relevant diagnostic Read code (see original study protocol in Supplementary Methods online) was recorded in a child’s medical records and/or when a prescription for asthma-related medication was issued (see original study protocol in Supplementary Methods online) in a child who had no wheezing/asthma diagnosis or related prescriptions previously recorded. Given the difficulty in distinguishing between wheezing disorders and asthma, especially in young children^19^, we considered an inclusive set of diagnostic codes. Medications included oral leukotriene receptor antagonists, and inhaled beta-2-agonists, cromoglycates, ipratropium bromide, and glucocorticoids. The first recording of wheezing/asthma or a related prescription was considered the index date. Children moving into a GP practice were considered prevalent cases if wheezing/asthma was recorded before or on the first day of registration with that practice.

RTIs, including both acute lower RTIs (LRTIs; including: bronchitis, bronchiolitis, pneumonia (including influenza pneumonia)) and acute upper RTIs (URTIs; including: otitis media, mastoiditis, rhinitis, sinusitis, pharyngitis, laryngitis, tonsillitis, epiglottitis, tracheitis, influenza (excluding pneumonia)), were identified using Read codes (see original study protocol in Supplementary Methods online for full list of diagnostic codes). Multiple consultations within a 14-day period were considered a single RTI episode, with the exception of LRTIs, which were allowed a preceding consultation for an URTI. As the majority of common childhood RTIs resolve within two weeks^20^, RTIs occurring beyond the 14-day period were considered new events.

The primary analysis was carried out for England, which had the largest population available for analysis. As secondary outcomes in England we considered the incidence of LRTIs and URTIs separately. Analyses for Scotland, Wales, and Northern Ireland were restricted to the two primary outcomes only.

### Data sources and handling

Data were obtained from CPRD (previously called the General Practice Research Database (GPRD)), which currently covers a representative 8.5% of the UK population^21^,^22^. We counted all incident wheezing/asthma diagnoses and RTI episodes between 1 January 1997 and 31 December 2012 occurring in children aged ≤12 years registered in practices who contributed ‘up-to-standard’ data at the time of the event (i.e. data meeting specific quality criteria as defined by CPRD)^23^. Incident wheezing/asthma and RTI counts were separately aggregated per month for each country. Separate counts for LRTIs and URTIs were also recorded.

The at-risk-population in a given month consisted of all children registered with a GP practice that contributed to up-to-standard data for at least part of that month. For analysis of wheezing/asthma diagnoses, prevalent cases were excluded from the denominator. We restricted our analyses to children aged ≤12 years in order to minimise potential confounding by active smoking.

### Statistical analyses

Monthly counts for each outcome were analysed in the statistical package R version 2.14.0, using the function ‘gamm’ from the library ‘mgcv’^24^. We used Poisson generalised additive mixed models (GAMMs) to calculate an incidence rate ratio (IRR) for each outcome, indicating the change in its incidence in the period after the introduction of smoke-free legislation compared to the period beforehand. We used GAMM models because their flexibility allows the effects of both linear and non-linear terms to be estimated, and autocorrelation between data points to be accounted for^24^.

Each model included variables specified *a priori* to account for differences in the number of days in each month, the number of days GP practices were open (i.e. excluding weekends and public holidays), and an offset term indicating the size of the at-risk-population. Smoke-free legislation was modelled using a dummy variable coded ‘0’ before its introduction in each country, and ‘1’ afterwards, thus allowing for an instantaneous (‘step’) change in the incidence of the outcome. Statistical significance was accepted at a two-tailed alpha of 0.05.

A backward selection procedure using a cut-off p-value of 0.05 was employed to allow for additional inclusion of the following variables: a thin plate spline or linear term to model any underlying time trends (using the effective degrees of freedom of the spline to assess whether a non-linear or linear term was most appropriate); a cyclic cubic spline to model seasonal variations; monthly mean temperature (average of the mean monthly maximum and minimum temperatures across all Meteorological Office weather stations in each country)^25^. Following initial analysis, in a minor deviation from the protocol, three additional covariates were considered to enable modelling of residual seasonality within the data: RTI consultation rate (in the model of wheezing/asthma incidence) and two dummy variables to represent school holidays. Relative troughs in the incidence of respiratory outcomes during school holiday periods are well recognised in both preschool and school-aged children^26^. We therefore defined a dummy variable coded ‘1’ in August of each year and ‘0’ in all other months to model the impact of the long summer holiday, and a second dummy to account for the Easter (April) and Christmas (December) holidays. These holidays are typically of two weeks’ duration, and could not be more accurately specified within our monthly data; we did not attempt to model the impact of the one-week ‘half-term’ holidays. Given the established association between RTIs and childhood wheezing disorders^27^, RTI incidence was included in the analyses of new wheezing/asthma diagnoses. This variable was log-transformed to normalise its distribution.

In addition, initial examination of the RTI count data suggested higher than usual counts in July 2009 which we hypothesised to be attributable to pandemic influenza; the highest GP consultation rates for influenza-like illness in UK sentinel practices occurred in week 29 (ending 19 July)^28^. Our preliminary models failed to adequately capture these values. We therefore defined a dummy variable for inclusion in our RTI models to model this peak pandemic period, coded ‘1’ in July 2009 and ‘0’ in all other months.

We observed overdispersion in the Poisson models, which we corrected using a quasi-Poisson model. Model residuals were examined to assess normality and identify any residual autocorrelation. Autoregressive or moving average terms were included in the model as appropriate, using the residual autocorrelation function and partial autocorrelation function to determine the order of these terms. The final selection of covariates for each model is displayed in Supplementary Table S1 online.

### Subgroup analyses

We performed pre-defined subgroup analyses by age group (0–4 years; 5–12 years) to assess potential differential effects of smoke-free legislation in preschool and school-age subgroups.

### Sensitivity analyses

Introduction of the Quality and Outcomes Framework (QOF) in April 2004 provided GPs with a financial incentive to record asthma diagnoses, which might be hypothesised to produce sudden changes in incidence rates. Therefore, we also ran our model for wheezing/asthma diagnoses in England on a restricted time series from April 2004 only, as defined *a priori*.

## Results

**Figure 1:**
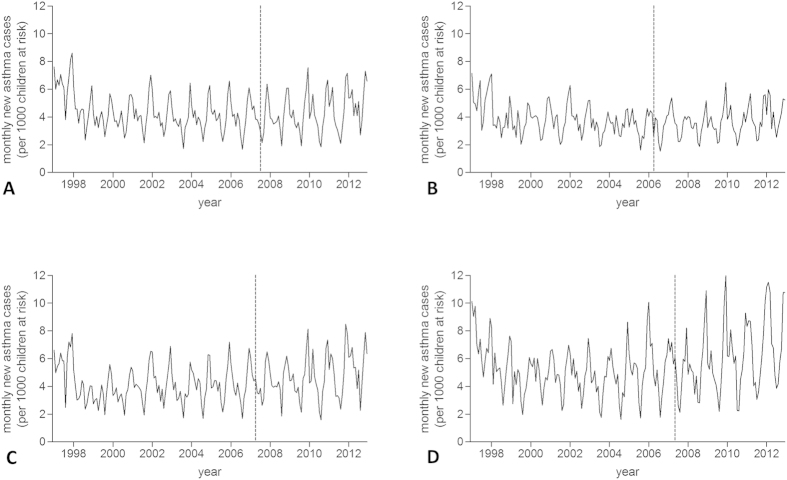
Incidence trends in new wheezing/asthma diagnoses. (**A**) England; (**B**) Scotland; (**C**) Wales; (**D**) Northern Ireland. Dashed line indicates introduction of smoke-free legislation.

**Figure 2:**
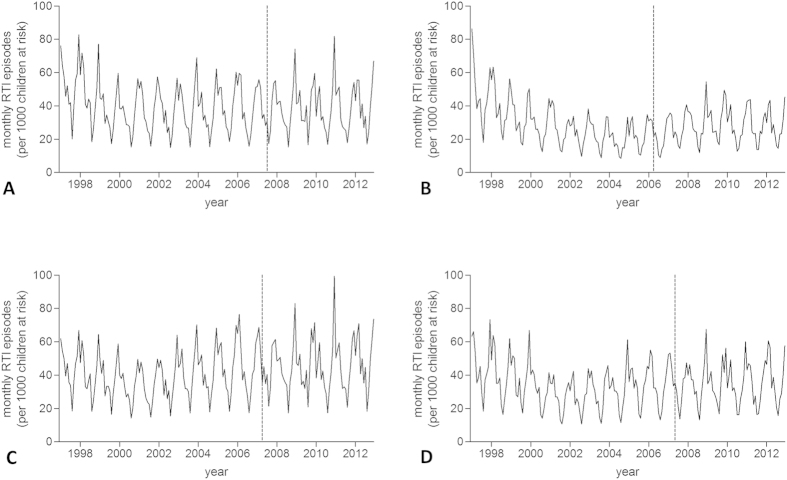
Incidence trends in new respiratory tract infection (RTI) diagnoses. (**A**) England; (**B**) Scotland; (**C**) Wales; (**D**) Northern Ireland. Dashed line indicates introduction of smoke-free legislation.

A total of 9,536,003 patient-years of data was analysed, during which 366,642 new wheezing/asthma diagnoses and 4,324,789 RTI episodes were observed (Table 1). 15,433 URTI consultations were followed by a LRTI episode within 14 days. Temporal patterns in the number of practices contributing data are given in Supplementary Fig. S1 online. Monthly rates of new wheezing/asthma diagnoses and RTIs for each country are displayed in Figs 1 and 2, respectively. Mean monthly wheezing/asthma incidences across the whole study period per 1,000 children at risk ranged from 3.7 for Scotland to 5.4 for Northern Ireland (overall mean 4.3). Comparable figures for RTIs ranged from 26.3 for Scotland to 42.1 for Wales (overall mean 37.8).

The introduction of smoke-free legislation in England was not associated with a statistically significant change in the number of new wheezing/asthma diagnoses in GP practices: IRR 0.94 (95% CI 0.81–1.09, p = 0.412). Similarly, no significant impact of smoke-free legislation on new diagnoses of wheezing/asthma was observed in Scotland, Wales, or Northern Ireland (Table 2).

There was no statistically significant change in the incidence rate of RTI episodes in the GP practice following introduction of smoke-free legislation in England (IRR 0.95 (95% CI 0.84–1.06), p = 0.399). Neither was a significant change observed in the other UK countries (Table 2).

**Figure 3:**
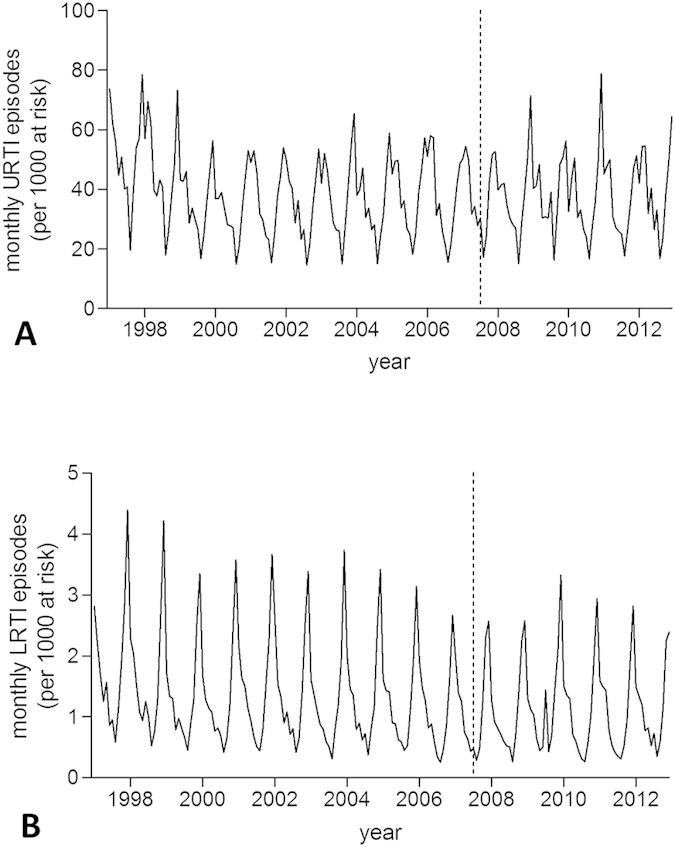
Incidence trends in upper and lower respiratory tract infection episodes in England. (**A**) upper respiratory tract infections (URTI); (**B**) lower respiratory tract infections (LRTI). Dashed line indicates introduction of smoke-free legislation. Note the different scales on the y-axis.

The vast majority of RTI episodes were URTIs (Table 1 and Fig. 3). There was no statistically significant impact of smoke-free legislation on either URTI episodes (IRR 0.95 (95% CI 0.86–1.06), p = 0.401) or LRTI episodes (IRR 0.96 (95% CI 0.81–1.15), p = 0.678) in separate analyses for England.

**Figure 4:**
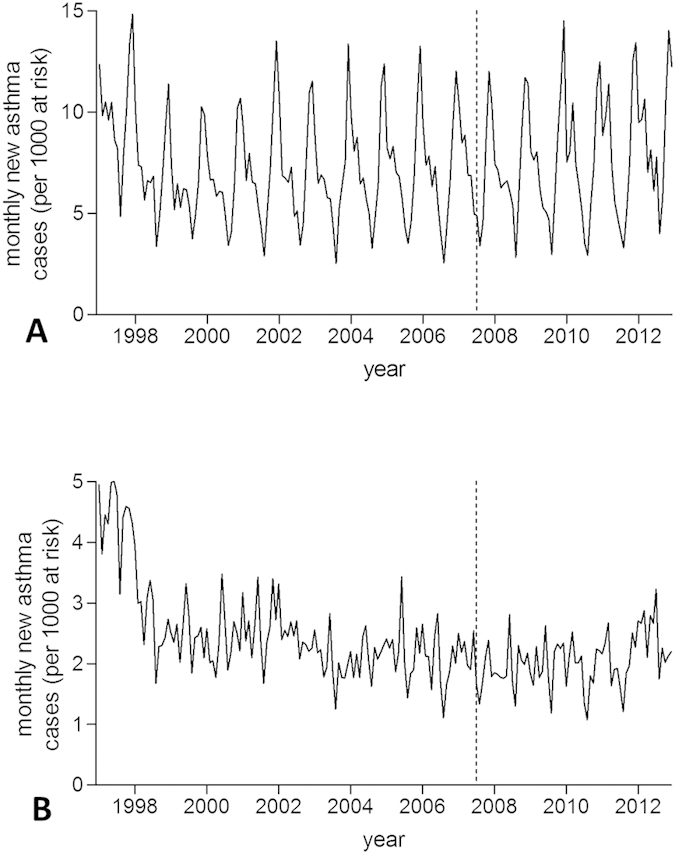
Incidence trends in new wheezing/asthma diagnoses in England according to age group. (**A**) 0–4 years; (**B**) 5–12 years. Dashed line indicates introduction of smoke-free legislation. Note the different scales on the y-axis.

**Figure 5:**
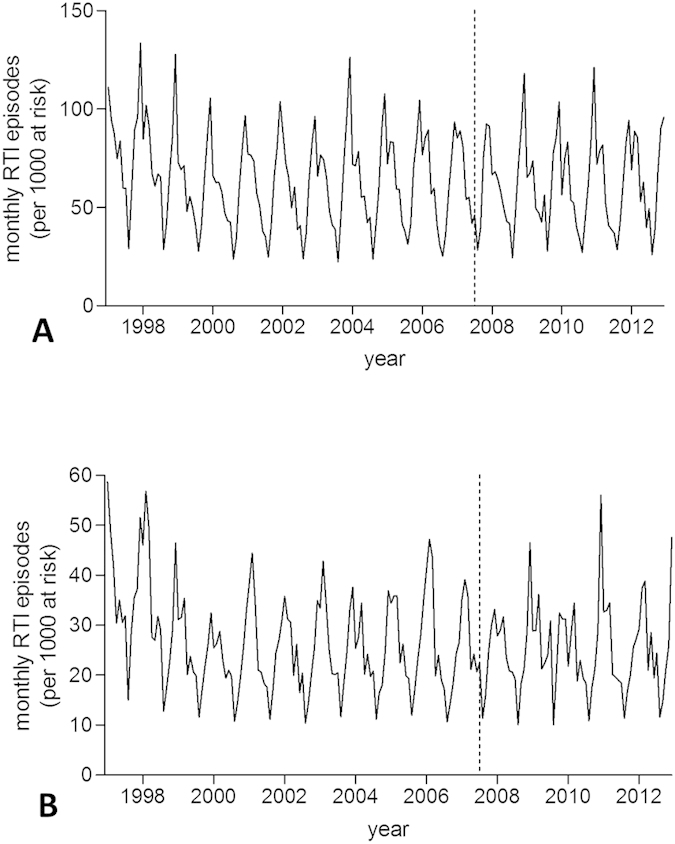
Incidence trends in respiratory tract infection (RTI) episodes in England according to age group. (**A**) 0–4 years; (**B**) 5–12 years. Dashed line indicates introduction of smoke-free legislation. Note the different scales on the y-axis.

New wheezing/asthma diagnoses and RTI episodes both occurred more frequently among preschool children than among school-age children (Figs 4 and 5). No significant impact of smoke-free legislation on either outcome was seen in England irrespective of age group (Table 3).

Restriction of the time series analysis to the period following the introduction of the QOF did not affect the impact estimation of smoke-free legislation on the incidence rate of new wheezing/asthma diagnoses in England, which remained non-statistically significant: IRR 0.93 (95% CI 0.84–1.02), p = 0.123.

## Discussion

In this large observational study we found no association between the introduction of smoke-free legislation and the number of new wheezing/asthma cases or RTI episodes among children presenting to a GP in any of the four countries in the UK.

Analysing over 350,000 new wheezing/asthma cases and more than four million RTI episodes over a 16-year period in four countries, this study is one of the largest evaluations of the impact of smoke-free legislation on child health ever undertaken^5^. It is unique in its focus on evaluation of GP consultations, as previous studies investigated hospital-based outcomes^5^. CPRD is the largest longitudinal GP database in the world, and its validity is well established^22^. We included only data that met pre-specified data quality criteria and used well-defined case definitions, using diagnostic and prescription data. We focused on children aged 12 and younger to minimise potential confounding by active smoking. The unique geographic coverage of CPRD furthermore allowed us to replicate the methodology in all four UK countries, where smoke-free legislation was introduced at different time points, further strengthening the findings.

This study also has a number of limitations. Inherent to the evaluation of national public health interventions, randomised allocation of the intervention was not possible^29^. We therefore employed an interrupted time series analysis design, which is among the strongest designs for evaluation of public health interventions^29^. Nonetheless, the observational design and lack of a control group are limitations of the current study that restrict causal inference. Minor modifications to the original study protocol, deemed necessary after initial examination of the CPRD data, were specified. This is important as changes in approach may affect study findings^30^.

A variety of statistical models have been used in the past to assess the association between smoke-free legislation and health outcomes^3^,^5^,^29^, and may result in different findings^5^,^31^. Particular strengths of the GAMM modelling approach include its ability to deal with issues that commonly complicate temporal data analysis, such as the need to account for underlying non-linear long-term and seasonal variations in the outcome, and temporal autocorrelation. However, the modelling approach did not allow us to take into account potential individual-level confounding or data dependency resulting from recurrent RTI events within individuals.

In interrupted time series analysis, it is assumed that all other factors influencing the outcome, including the composition of the study population, remain unchanged. With patients and GP practices entering and leaving the database over time, consequential temporal variation in population characteristics may have introduced bias. We are unaware of a systematic assessment of the potential limitations of using interrupted time series GAMM in this setting. Temporal constancy is also violated if the degree of recording of the outcome changes over time. A sensitivity analysis suggested no confounding by the 2004 introduction of QOF. No abrupt incidence rate deviations that could suggest temporal changes in recording were present in any outcome and temporal patterns of GP consultations generally paralleled those of asthma and RTI hospitalisations^6^,^32^. To minimise the risk of under-recording of wheezing/asthma, we included medication prescriptions in the definition.

Reliable case definition in general practice is challenging. This is particularly true for asthma, which is a difficult diagnosis, especially among preschool children^19^. Our highly inclusive list of diagnostic codes may have led to over-diagnosis, and it is possible that stricter case definitions would have generated different impact estimations. Although the overall validity of RTI diagnoses in CPRD has been shown to be very high at 97.2% (95%CI 85.5–99.9)^22^, we are unaware of similar data for asthma^22^. Wheezing/asthma cases and RTI episodes may have been missed because they presented primarily in secondary care, although their contribution is likely to be minimal. A particular issue concerned the identification of prevalent asthma cases. Previous research using GPRD data showed that patients were more likely to be diagnosed with asthma in the first three months after their GP practice becoming up-to-standard, probably due to misclassification of prevalent cases as incident cases^33^. In our study this is likely to be of only minor importance as most children entered the database shortly after birth.

The effect estimates in our study had wide confidence intervals. Using the standard error of the IRRs derived from our models we retrospectively estimated the minimum effect size that could be detected as statistically significant at approximately 15–20%^34^, a larger decline than our point estimates. The primary determinant of the uncertainty around our estimates and thus of the reduced power, despite a very large study population and extensive study period, is the ability of GAMM to capture the month-by-month variability in the time series. Despite additional efforts to adequately model this, an important degree of unexplained variation remained, hence the wide confidence intervals observed. In an unplanned sensitivity analysis (not shown), analysing weekly rather than monthly data resulted in similar effect sizes and no improvement in power. Temporal variation in meteorological and environmental factors, RTI epidemics, and demographic changes due to the dynamic character of the cohort not accounted for in our models may contribute to the unexplained variation.

Many of the issues described above could potentially be addressed by performing additional sensitivity analyses. As outlined in our study protocol, given the number of planned analyses and the corresponding risks associated with multiple testing, we restricted our analyses to those presented.

Unequivocal evidence supports the link between tobacco smoke exposure and adverse respiratory outcomes among children and adults, including RTIs and the development and exacerbation of chronic lung disorders such as asthma and chronic obstructive pulmonary disease^7^,^8^,^9^,^12^. In line with these findings, meta-analyses show that smoke-free legislation is associated with reductions in hospital attendance for respiratory infections among adults, and for asthma among both children and adults^3^,^5^. These effects are likely attributable to reductions in second-hand smoke exposure in the public as well as the home environment^2^,^5^. In England, between 2006 and 2008 the proportion of smoke-free homes among children with smoking parents increased from 36% to 48% (p < 0.001), and the proportion of children with undetectable salivary cotinine, a marker of SHS exposure, increased from 34% to 41% (p < 0.001)^35^. Three UK studies evaluated the impact on paediatric respiratory health^6^,^32^,^36^, demonstrating substantial reductions in asthma hospitalisations following smoke-free legislation in England (immediate: IRR 0.91, 95% CI 0.89–0.93; annual: IRR 0.97, 95% CI 0.96–0.98) and in Scotland (gradual: IRR 0.82, 95% CI 0.78–0.85), as well as in RTI admissions in England (immediate: IRR 0.965, 95% CI 0.953–0.977; annual: IRR 0.995, 95% CI 0.991–0.999). Retrospective power calculation demonstrated that our study likely lacked power to detect differences in the same order of magnitude. Adequate comparison of these studies with our work is complicated by methodological differences (e.g. evaluation of hospitalisations versus GP consultations; different coding systems; the use of negative binomial regression versus GAMM; the evaluation of gradual versus immediate incidence changes; and model adjustment for demographic variables)^6^,^32^,^36^.

It is possible that indeed no significant reduction in RTI consultations and new wheezing/asthma cases in general practice followed the introduction of smoke-free legislation in England, as our findings suggest. For RTIs one could speculate that smoke-free laws preferentially reduce the incidence of severe and complicated cases, which are more likely to require hospitalisation^3^,^6^, rather than overall RTI incidence. This fits well with the recognised positive association between SHS exposure and RTI severity among children^37^,^38^. As for asthma, smoke-free legislation may reduce the frequency of exacerbations as indicated by hospital attendance^5^, rather than the actual incidence of new wheezing/asthma diagnoses, as evaluated in the current study. In support of this concept, US children living in regions with a smoking ban were shown to have lower rates of asthma symptoms, but not of current asthma, compared to those living in regions without a ban^39^. We did not attempt to assess the contribution of asthma exacerbations in our study, due to uncertainty regarding their reliable differentiation from regular asthma visits.

The current study leaves a number of questions unanswered. Variations in case definition and sensitivity analyses to explore data validity issues should be considered, as well as the additional inclusion of older age groups and non-respiratory disease outcomes that can be expected to benefit from smoke-free legislation^2^,^3^,^5^. Parallel evaluations of primary and secondary care outcomes in the same population are necessary to more precisely identify the areas where smoke-free environments exert their primary benefit. As the largest burden of morbidity and mortality associated with respiratory disorders lies within low- and middle-income countries, the lack of health impact studies of smoke-free environments in these regions needs to be addressed^5^.

In conclusion, despite strong existing evidence for reductions in severe adverse early life health outcomes following smoke-free legislation, no significant changes in the incidence of GP diagnoses of wheezing/asthma or RTIs among children were demonstrated in this large study in four UK countries.

## Additional Information

**How to cite this article**: Been, J. V. *et al.* Smoke-free legislation and the incidence of paediatric respiratory infections and wheezing/asthma: interrupted time series analyses in the four UK nations. *Sci. Rep.* **5**, 15246; doi: 10.1038/srep15246 (2015).

## Supplementary Material

Supplementary Information

## Figures and Tables

**Table 1:** Event counts and patient-years observed by diagnosis, country, and age group.

Country, age group	Asthma			Respiratory tract infections						
	Events (n)	Patient-years observed (n)	Mean incidence per 1,000 patient-months	All RTI events (n)	URTI events (n)	LRTI events (n)	Patient-years observed (n)	Mean RTI incidence per 1,000 patient-months	Mean URTI incidence per 1,000 patient-months	Mean LRTI incidence per 1,000 patient-months
England										
0–4 years	197,942	2,220,194	7.4	2,030,265	1,949,508	92,166	2,679,568	63.1	60.6	2.9
5–12 years	96,092	3,500,493	2.3	1,525,504	1,503,407	23,467	4,940,896	25.7	25.4	0.4
Total	294,034	5,720,687	4.3	3,555,769	3,452,915	115,633	7,620,464	38.9	37.8	1.3
Scotland										
0–4 years	18,905	252,940	6.2	150,990	144,936	6,870	295,511	42.6	40.9	1.9
5–12 years	10,372	408,272	2.1	118,462	116,979	1,598	557,239	17.7	17.5	0.2
Total	29,277	661,212	3.7	269,452	261,915	8,468	852,750	26.3	25.6	0.8
Wales										
0–4 years	19,067	206,206	7.7	200,546	191,956	9,697	250,071	66.8	64.0	3.2
5–12 years	9,344	334,719	2.3	165,071	162,645	2,559	473,702	29.0	28.6	0.5
Total	28,411	540,925	4.4	365,617	354,601	12,256	723,773	42.1	40.8	1.4
Northern Ireland										
0–4 years	10,681	87,983	10.1	75,931	72,843	3,526	115,320	54.9	52.6	2.5
5–12 years	4,239	140,867	2.5	58,020	57,196	869	223,695	21.6	21.3	0.3
Total	14,920	228,850	5.4	133,951	130,039	4,395	339,015	33.0	32.0	1.1
All countries										
0–4 years	246,595	2,770,073	7.4	2,457,732	2,359,243	112,259	3,340,470	61.3	58.9	2.8
5–12 years	120,047	4,394,408	2.3	1,867,057	1,840,227	28,493	6,195,533	25.1	24.8	0.4
Total	366,642	7,151,674	4.3	4,324,789	4,199,470	140,752	9,536,003	37.8	36.7	1.2

RTI = respiratory tract infection; URTI = upper respiratory tract infection; LRTI = lower respiratory tract infection.

**Table 2:** Impact of smoke-free legislation on primary outcomes.

Country	Asthma			Respiratory tract infections		
	IRR	95% CI	p-value	IRR	95% CI	p-value
England	0.94	0.81–1.09	0.412	0.95	0.86–1.06	0.399
Scotland	0.99	0.83–1.19	0.946	0.96	0.83–1.12	0.620
Wales	1.09	0.89–1.35	0.406	0.97	0.86–1.09	0.641
Northern Ireland	0.96	0.76–1.22	0.745	0.90	0.79–1.03	0.132

See Supplementary Table S1 online for details of the variables included in each model. IRR = incidence rate ratio; CI = confidence interval.

**Table 3:** Impact of smoke-free legislation in England on primary outcomes by age group.

Age group	Asthma			Respiratory tract infections		
	IRR	95% CI	p-value	IRR	95% CI	p-value
0–4 years	0.96	0.84–1.10	0.580	0.97	0.88–1.08	0.621
5–12 years	0.92	0.77–1.10	0.367	0.90	0.78–1.03	0.114

See Supplementary Table S1 online for details of the variables included in each model. IRR = incidence rate ratio; CI = confidence interval.
