# Supplementary material for: Smoke-free legislation and the incidence of paediatric respiratory infections and wheezing/asthma: interrupted time series analyses in the four UK nations
Source: Sci Rep. 2015 Oct 14;5:15246. doi: 10.1038/srep15246 (PMC4604467; doi:10.1038/srep15246)
Supplement: Supplementary Information [file srep15246-s1.pdf]

Supplementary Information

Smoke-free legislation and the incidence of paediatric respiratory infections and wheezing/asthma: interrupted time series analyses in the four UK nations

Jasper V Been, Lisa Szatkowski, Tjeerd-Pieter van Staa, Hubert G Leufkens, Onno C van Schayck, Aziz Sheikh, Frank de Vries, Patrick Souverein

Contents:

Supplementary Figure S1. Number of GP practices contributing data to CPRD

Supplementary Table S1. Covariates included in models via backward selection process

Supplementary Methods. Original study protocol

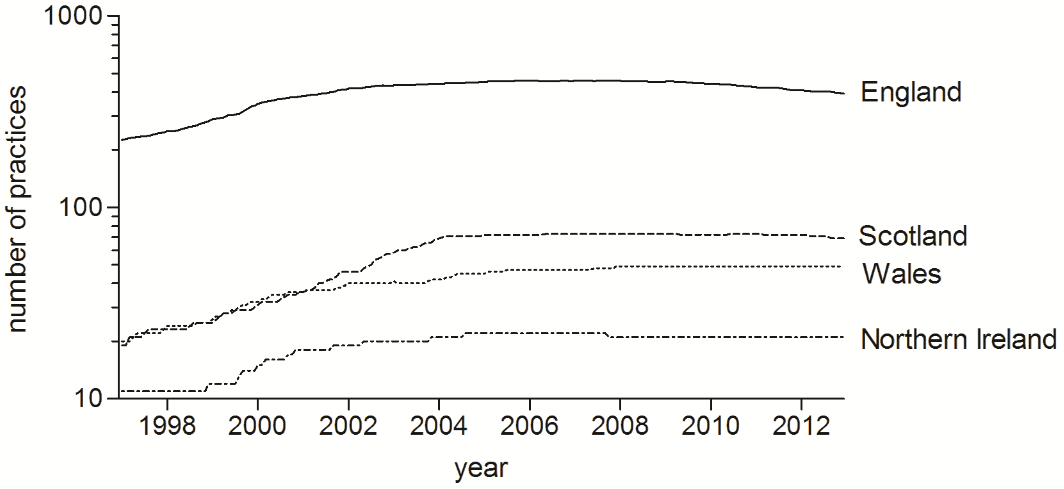

Supplementary Figure S1. Number of GP practices contributing data to CPRD. Note logarithmic scale on y-axis.

Supplementary Table S1: Covariates included in models via backward selection process

Model	Temperature	August holiday	April + December holidays	RTI consultation rate	Pandemic influenza	Autocorrelation term
New asthma diagnoses						
England (all ages)		×	×	×		MA(1)
England (0-4 years)		×	×	×		MA(1)
England (5-12 years)		×	×	×		MA(2)
England (QOF sensitivity analysis; all ages)		×	×	×		MA(1)
Scotland				×		MA(1)
Wales		×	×	×		MA(1)
Northern Ireland		×	×	×		MA(1)
Respiratory tract infections						
England (all ages)	×	×	×		×	MA(1)
England (0-4 years)	×	×	×		×	MA(2)
England (5-12 years)	×	×	×		×	MA(1)
Scotland		×	×			MA(1)
Wales		×	×			MA(1)
Northern Ireland		×	×			MA(1)
Upper respiratory tract infections						
England (all ages)	×	×	×		×	MA(1)
Lower respiratory tract infections						
England (all ages)		×	×		×	MA(1)

All models furthermore included a thin plate spline to account for the underlying time trend, a cubic spline to account for seasonality, terms to account for variations in the number of days in a month and the number of days GP practices were open (there was no strong correlation between these), and an offset variable to indicate the number of patients at risk. In the QOF sensitivity analysis the underlying time trend was non-linear. RTI = respiratory tract infection; MA=moving average; QOF=Quality and Outcomes Framework

Supplementary Methods:

ORIGINAL STUDY PROTOCOL

TITLE

Smoke-free legislation and childhood respiratory infections and asthma in primary care: an explorative study

AUTHORS

Jasper V. Been; Lisa Szatkowski; Tjeerd-Pieter van Staa, Patrick Souverein; Hubert G.M. Leufkens; Constant P. van Schayck; Aziz Sheikh; Frank de Vries

ABBREVIATIONS

CPRD	Clinical Practice Research Datalink
HES	Hospital Episode Statistics
ICD	International Classification of Diseases
IMD	Index of Multiple Deprivation
QOF	Quality and Outcomes Framework
SES	socioeconomic status
SHS	second-hand smoke exposure
WHO	World Health Organization

LAY SUMMARY

Exposure to other people's tobacco smoke (second hand smoke; SHS) conveys an important health risk. Children are particularly vulnerable to adverse effects of SHS and are unable to influence their own degree of exposure. To protect people from SHS the World Health Organization (WHO) recommends implementation of smoke-free laws. Evidence is now emerging that smoke-free laws improve paediatric health, for example by decreasing hospital admissions for asthma. The effect of smoke-free legislation on the occurrence of asthma and respiratory infections among children that present to the GP has never been studied. We will study this effect of smoke-free across the UK, where smoke-free legislation was introduced at different time points in each of the four countries. Trends in the occurrence of asthma and respiratory infections will be investigated from 1997 to 2012 using data from the Clinical Practice Research Datalink (CPRD), which currently covers about 5-10% of GP practices in the UK. Effect of other factors that might influence trends in these diseases will be taken into account. To the best of our knowledge this is the first explorative study to assess the effect of smoke-free legislation on infant and child health in primary care and as such may help inform the development and implementation of additional measures to protect infants and children around the world from adverse effects of SHS.

OBJECTIVE, SPECIFIC AIMS AND RATIONALE

We aim to describe the association between introduction of smoke-free legislation in different countries in the UK and incidence changes in asthma and respiratory tract infections (RTIs) in primary care among children aged 12 and younger.

BACKGROUND

Tobacco use kills more than five million people annually making it the leading global cause of preventable death [1]. It is estimated that exposure to other people's tobacco smoke ("second-hand smoke" (SHS)) kills an additional 600.000 people worldwide each year, including 166.000 children under 15 years of age [1, 2]. Among non-smoking adults, SHS exposure also increases the risk of developing asthma, lung cancer and ischaemic heart disease [2]. In an attempt to reduce this substantial burden on passive smokers, the World Health Organization (WHO) has recommended that smoke-free indoor public environments are enforced through national legislation and that educational strategies are pursued in parallel to reduce SHS exposure in the home [3]. Studies have since shown that smoking bans effectively reduce SHS exposure, and through doing so reduce respiratory and sensory symptoms, sudden cardiac arrest, and admissions for acute myocardial infarction as well as associated mortality [4-6].

As developing individuals, children are particularly vulnerable to the negative effects of SHS and they are unable to influence their own degree of exposure. Currently over 40% of children worldwide are regularly exposed to SHS [1]. SHS exposure both before and after birth increases the risk of adverse health outcomes in early life, and has been shown to increase the risk of stillbirth, preterm birth, low birth weight, and RTIs as well as asthma in childhood [7-12]. Children are thus particularly likely to benefit from smoke-free laws. Indeed, recent evaluations of the 2006 Scottish smoking ban showed reductions in preterm birth and low birth weight, as well as hospital admissions for asthma among children after its introduction [13, 14]. The latter finding has recently been confirmed in a similar study in England [15].

Knowledge gap

It is important that evaluations of smoke-free laws are reproduced in several countries where smoke-free laws were introduced at different time points given concerns that the evidence inevitably comes from quasi-experimental studies, which are inherently at high risk of bias [16]. Furthermore, an evaluation of child health effects of smoke-free legislation has hitherto been mainly based on the study of hospitalisations. We propose to build on this work by using data from the Clinical Practice Research Datalink (CPRD) database, which currently covers 5-10% of GP practices across the UK. This will provide an important community-based perspective to the current evidence base. From CPRD we will obtain disease trends for paediatric asthma and RTIs in primary care. Smoke-free laws were introduced at different time points in Scotland (March 26, 2006), Wales (April 2, 2007), Northern Ireland (April 30, 2007), and England (July 1, 2007), facilitating comparison of associated disease incidence trend changes among countries. As such the proposed study will serve to further inform the development and

implementation of global policy and strategies to further reduce SHS exposure in a particularly vulnerable population.

We hypothesise that introduction of smoke-free laws across the UK is associated with decreases in the incidence of asthma and RTIs in primary care among children aged 12 and younger.

STUDY TYPE

Hypothesis generating / descriptive study

STUDY DESIGN

Interrupted time series using generalised additive mixed models (GAMM)

SAMPLE SIZE / POWER CALCULATION

Sample size calculation for time-oriented analyses is complicated given the complexity of the models. Formal power calculations are non-existent for GAMM models. A previous epidemiological evaluation of the 2007 English smoking ban among children <15 years of age showed a significant sudden drop in asthma hospitalisations of 9% (95% confidence interval (CI) 7-11%) and a subsequent annual drop of 3% (95% CI 2-4%) among children <15 years of age [15]. A similar evaluation of the 2006 Scottish smoking ban demonstrated an 18% (95% CI 15-22%) annual reduction in asthma hospitalisations among children <15 years of age [13]. To the best of our knowledge no prior studies have evaluated the effect of smoke-free legislation on paediatric RTIs. Meta-analyses of observational studies show that among children the association between second-hand smoke exposure and RTIs is of greater magnitude than that of smoke exposure and asthma [2, 10-12]. Any effect of smoke-free legislation on paediatric RTIs is therefore likely to be larger than that estimated for asthma. Considering the much higher incidences of asthma and RTIs in primary care as opposed to secondary care and the larger time span of our proposed study, we are confident that our study is adequately powered to detect statistically significant and clinically relevant drops in incidences of the proposed outcomes, even though CPRD holds primary health care data for 'only' 5-10% of the UK population.

STUDY POPULATION

The study population will consist of all children aged 12 or younger registered with a CPRD practice for at least part of the study period (January 1st 1997 to December 31st 2012). The age cut-off is selected in an attempt to minimise the potential confounding effect of self-smoking.

SELECTION OF COMPARISON GROUP(S) OR CONTROLS

An interrupted time series analysis does not employ a comparison or control group in the usual sense of this term. GAMM models will be used to model monthly counts of the number of children diagnosed with asthma or a RTI from January 1997 to December 2012 taking into account the number of children registered in CPRD during the month and who are thus 'at risk' of developing the outcome. An incidence rate ratio (IRR) with 95% CI will be calculated to quantify any change in the incidence of asthma and RTI in the months after the introduction of smoke-free legislation compared to the months beforehand. The model will account for any underlying long-term trend in asthma/RTI incidence (e.g. a long-term increase or decrease), as well as seasonal variations in the outcome.

EXPOSURES, OUTCOMES AND COVARIATES

Exposure

Every patient will be followed from the start of valid data collection or 1 January 1997, whichever comes latest, until the end of valid data collection, the end of the study period, or until 31 December of the year before the child becomes 13 years old. If the month of birth is available for a child, he/she will be followed until the latest day of the month before he/she becomes 13 years old. We have found however that month of birth is not available for about 58% of children in the database. For these children the date of first registration with a CPRD practice will be taken as a proxy for date of birth if they occur in the same year. If birth year precedes the year of registration, July 1 will be assigned as the birth month.

The exposure in this study is the intervention under investigation, namely exposure to a national smoking ban.

Outcomes

1. Asthma

Incident asthma diagnoses in primary care will be evaluated, using the following definition:

- recording of a medical code indicative of a wheezing disorder (Appendix 1)
- and/or the issue of a prescription of asthma-related medication (BNF chapters 3.1.1; 3.1.2; 3.1.4; 3.2; 3.3.1; 3.3.2; a detailed list is given in Appendix 2)
- and no prior recording of an asthma diagnosis or prescription of asthma-related medication (this includes the period preceding and containing the first day of valid (e.g. 'up to standard') data collection).

The earliest recording wheezing / asthma related medication defines the index date.

A new asthma diagnosis is thus made when any diagnostic code for asthma and/or any prescription of medication used for asthma are recorded for the first time in a particular patient. A full list of Read codes used for asthma diagnosis is provided in Appendix 1. A list of medications considered to be indicative of asthma for the purpose of this study can be provided upon request.

An inclusive definition of asthma is used for this study given the clinical heterogeneity of wheezing disorders in childhood and the inability to reliably distinguish between different wheezing phenotypes using routine health care data. Furthermore, most if not all wheezing phenotypes are expected to be affected by SHS exposure and are therefore potentially sensitive to an effect of smoke-free legislation.

2. Respiratory infection

The primary outcome will be all acute RTIs in primary care, using the following definition:

- Primary outcome:
    - recording of a medical code indicative of a RTI (Appendix III).
    - and no recording of an RTI during the 14 days preceding the diagnosis

The primary outcome will involve all acute upper and lower RTIs. A full list of Read codes used for respiratory tract infection diagnoses is provided as Appendix 2. A respiratory infection is considered new when being registered at least 14 days after any prior respiratory infection in order to exclude repeated GP visits for the same RTI.

Secondary outcomes will be all acute upper RTIs and all acute lower RTIs using the following definitions:

- Secondary outcomes:
    - Upper RTIs:
        - recording of a medical code indicative of an upper RTI (Appendix III)
        - and no recording of any RTI during the 14 days preceding the diagnosis
    - Lower RTIs:
        - recording of a medical code indicative of a lower RTI (Appendix III).
        - and no recording of a lower RTI during the 14 days preceding the diagnosis

Covariates

In GAMM modelling data are evaluated at aggregate level producing a rate ratio based on case counts. GAMM models therefore do not allow incorporation of individual-level covariates. Time series modelling is based on the assumption that the population structure does not change over time. Although this is expected to hold for most covariates, a change in age structure of the population over time may be expected given the increasing birth rate in the UK. Given the fact that both asthma and RTIs are more common among younger children, this may produce a spurious increasing trend in both outcomes over time. We will therefore perform a sensitivity analysis to assess the effects of the smoking ban among age subgroups, as discussed below.

National-level mean monthly temperature will furthermore be included as a predictor variable in each model, using data freely available from the UK Meteorological Office.

DATA / STATISTICAL ANALYSIS PLAN

Production of incidence rates

For every month, a denominator population will be defined. The denominator population consists of all patients aged 12 years and under who are enrolled on the 15th day of that month in CPRD practices that contribute up-to-standard data. For each month we will identify the number of children with each of our outcomes. We will calculate incidence rates and plot these over time in order to visualise obvious changes in temporal patterns.

Model description

We will use GAMM to model monthly counts of the number of children with each outcome from January 1997 to December 2012 taking into account the number of children registered in CPRD during the month and who are thus 'at risk' of developing the outcome. An incidence rate ratio with 95% confidence interval will be calculated to quantify any change in the incidence of asthma and RTI in the months after the introduction of smoke-free legislation compared to the months beforehand. The model will account for any underlying long-term trend (linear or non-linear, as appropriate) in asthma/RTI incidence (e.g. a long-term increase or decrease), as well as seasonal variations in the outcome (using a cyclic cubic spline). Model residuals will be checked for any remaining autocorrelation, which will be accounted for using autoregressive and/or moving average terms if necessary. National-level mean monthly temperature will also be included as a predictor variable in each model. The primary analysis will be performed in England and will involve both primary and secondary outcomes. Secondary analyses will separately be performed in a similar manner - but for primary outcomes only - in Northern Ireland, Scotland, and Wales. All analyses will be performed using the R statistical software package.

Validity issues

A number of potential threats to validity exist within this study. Given the multitude of primary and secondary analyses as well as potential validity threats, we will not perform separate sensitivity analyses to address these. Instead, these issues will be outlined and discussed in the report, with a scope of potentially addressing them in future work. The following potential validity threats are considered:

1. Validity of recording

An important assumption of time series analysis is that the quality and degree of recording are constant over time. Quality criteria will be applied as discussed previously. A theoretical threat to the consistency of the degree of recording is the 1 April 2004 introduction of the Quality and Outcomes Framework (QOF) which provides GPs with an incentive to record asthma diagnoses (not RTIs). Recording changes are expected to produce sudden changes in incidence rates that cannot be explained by external factors. Such changes are typically not present in databases measuring the same outcome using a different method. We will perform a basic assessment of this issue by visually comparing incidence plots obtained in this study to incidence rates of hospitalisations for asthma (as derived from HES and published by Millett et al [15]), as well as RTIs (as derived from HES, unpublished data; ClinicalTrials.gov NCT01920165).

2. Data dependency

In contrast to the asthma outcome, RTI diagnoses are allowed to occur recurrently within the same individual. Such intra-individual RTIs will have a greater level of interdependency than inter-individual RTIs. As GAMM models work with stratum-specific counts, they cannot account for this data dependency. Previous similar studies using hospital department visits and hospitalisations as outcomes experienced the same validity threat, which was not specifically addressed in any of the studies as the consequential risk of bias was deemed to be low [15, 17, 18].

3. Age

As mentioned previously, a change in age composition of the study cohort over time may result in spurious incidence changes of the outcomes of interest. As such changes occur gradually, this issue will in part be accounted for by accounting for underlying temporal trends in the models.

4. Pneumococcal vaccination

On September 4, 2006 the pneumococcal conjugate vaccine (PCV) was introduced into the UK childhood immunisations schedule at 2, 4, and 13 months of age. Children born from September 2004 were included in a catch-up programme. The relative contribution of true pneumococcal infections to the total burden of admissions for RTIs, the majority of which is likely to be of viral aetiology, is expected to be small. We assessed this issue in preliminary sensitivity analyses on childhood RTI admission data in England as obtained from HES, 2001-2012 (ClinicalTrials.gov NCT01920165). These demonstrate that estimation of the impact of smoke-free legislation was not influenced by accounting for PCV introduction. As the primary aim of PCV is reducing severe pneumococcal disease, which is likely to represent a very small proportion of all RTIs presenting to the GP, consequential bias of PCV introduction is likely to be negligible in the current study.

5. Asthma definition

The primary outcome definition may result in misclassification of prevalent asthma cases entering the database as ‘new’ (incident) cases upon their first recorded visit for asthma or first prescription after database entry. This has been shown to be a particular problem for subjects entering a practice that is already in the database (e.g. after the up-to-standard date for that practice [19]. As most children will enter a new practice by being born rather than moving house and the cohort is dynamic, with new patients entering the database continuously, potential consequential bias is expected to be small.

6. RTI definition

Using the primary definition, RTIs are considered incident when not preceded by another RTI in the 14 days preceding the event. This pragmatic cut-off may potentially introduce bias in two directions: 1. Mild upper RTIs may follow each other up at <14 day intervals, resulting in underestimation of the true incidence of ‘new’ RTIs; and 2. Chronic RTI disease episodes may extend beyond the 14-day period thus resulting in overestimation of the true incidence when the GP is visited for the same infection at a >14 day interval. This will however only introduce bias if the likelihood of any of these two situations occurring, changes over time. This is considered unlikely and the consequential bias is therefore expected to be low in this study.

7. Denominator definition

A very small proportion of patients will contribute to only part of the month, as they enter or leave the database during that month. In prior studies evaluating temporal smoking rate patterns, sensitivity analyses excluding such patients have shown negligible changes in outcome estimations.

PATIENT / USER GROUP INVOLVEMENT

We will liaise with members of The Netherlands Asthma Foundation.

LIMITATIONS OF THE STUDY DESIGN, DATA SOURCES AND ANALYTIC METHODS

The main limitation of this explorative study will be that a causal interpretation of the findings is not possible. For example, other policy changes may have taken place at the same time as the introduction of smoke-free legislation, which themselves may have led to changes in the outcomes of interest. However, interrupted time series analysis is among the strongest observational designs to infer possible causation where a randomised controlled trial is not ethical or feasible, as is the case with national smoke-free legislation. We will fully discuss the limitations of our analysis when preparing our work for publication. Other limitations have been discussed in the section “Validity issues” above.

PLANS FOR DISSEMINATING AND COMMUNICATING STUDY RESULTS

We plan to present our findings at scientific meetings and publish the results in a peer-reviewed scientific journal.

REFERENCES

1. WHO. WHO report on the global tobacco epidemic, 2009. Implementing smoke-free environments; 2009.

2. Oberg M, Jaakkola MS, Woodward A, Peruga A, Pruss-Ustun A. Worldwide burden of disease from exposure to second-hand smoke: a retrospective analysis of data from 192 countries. Lancet. 2011; 377(9760): 139-46.

3. WHO. Protection from exposure to second-hand smoke. Policy recommendations. 2007.

4. Callinan JE, Clarke A, Doherty K, Kelleher C. Legislative smoking bans for reducing secondhand smoke exposure, smoking prevalence and tobacco consumption. Cochrane database of systematic reviews (Online). 2010; (4): CD005992.

5. de Korte-de Boer D, Kotz D, Viechtbauer W, van Haren E, Grommen D, de Munter M, et al. Effect of smoke-free legislation on the incidence of sudden circulatory arrest in the Netherlands. Heart (British Cardiac Society). 2012; 98(13): 995-9.

6. Villalbi JR, Sanchez E, Benet J, Cabezas C, Castillo A, Guarga A, et al. The extension of smoke-free areas and acute myocardial infarction mortality: before and after study. BMJ open. 2011; 1(1): e000067.

7. Flenady V, Koopmans L, Middleton P, Froen JF, Smith GC, Gibbons K, et al. Major risk factors for stillbirth in high-income countries: a systematic review and meta-analysis. Lancet. 2011; 377(9774): 1331-40.

8. Leonardi-Bee J, Smyth A, Britton J, Coleman T. Environmental tobacco smoke and fetal health: systematic review and meta-analysis. Archives of disease in childhood. 2008; 93(5): F351-61.

9. Salmasi G, Grady R, Jones J, McDonald SD. Environmental tobacco smoke exposure and perinatal outcomes: a systematic review and meta-analyses. Acta obstetricia et gynecologica Scandinavica. 2010; 89(4): 423-41.

10. Jones LL, Hashim A, McKeever T, Cook DG, Britton J, Leonardi-Bee J. Parental and household smoking and the increased risk of bronchitis, bronchiolitis and other lower respiratory infections in infancy: systematic review and meta-analysis. Respiratory research. 2011; 12: 5.

11. Jones LL, Hassanien A, Cook DG, Britton J, Leonardi-Bee J. Parental smoking and the risk of middle ear disease in children: a systematic review and meta-analysis. Archives of pediatrics & adolescent medicine. 2012; 166(1): 18-27.

12. Burke H, Leonardi-Bee J, Hashim A, Pine-Abata H, Chen Y, Cook DG, et al. Prenatal and passive smoke exposure and incidence of asthma and wheeze: systematic review and meta-analysis. Pediatrics. 2012; 129(4): 735-44.

13. Mackay D, Haw S, Ayres JG, Fischbacher C, Pell JP. Smoke-free legislation and hospitalizations for childhood asthma. The New England journal of medicine. 2010; 363(12): 1139-45.

14. Mackay DF, Nelson SM, Haw SJ, Pell JP. Impact of Scotland's Smoke-Free Legislation on Pregnancy Complications: Retrospective Cohort Study. PLoS medicine. 2012; 9(3): e1001175.

15. Millett C, Lee JT, Laverty AA, Glantz SA, Majeed A. Hospital admissions for childhood asthma after smoke-free legislation in England. Pediatrics. 2013; 131(2): e495-501.

16. Freemantle N, Wood J, Crawford F. Evidence into practice, experimentation and quasi experimentation: are the methods up to the task? Journal of epidemiology and community health. 1998; 52(2): 75-81.

17. Mackay D, Haw S, Ayres JG, Fischbacher C, Pell JP. Smoke-free legislation and hospitalizations for childhood asthma. N Engl J Med. 2010; 363(12): 1139-45.

18. Rayens MK, Burkhart PV, Zhang M, Lee S, Moser DK, Mannino D, et al. Reduction in asthma-related emergency department visits after implementation of a smoke-free law. J Allergy Clin Immunol. 2008; 122(3): 537-41 e3.

19. Lewis JD, Bilker WB, Weinstein RB, Strom BL. The relationship between time since registration and measured incidence rates in the General Practice Research Database. Pharmacoepidemiol Drug Saf. 2005; 14(7): 443-51.

Appendix 1: READ codes for asthma

663	Asthma sometimes restricts exercise
1737	Wheezing
1737.11	Wheezing symptom
1780	Aspirin induced asthma
1781	Asthma trigger - pollen
1782	Asthma trigger - tobacco smoke
1783	Asthma trigger - warm air
1784	Asthma trigger - emotion
1785	Asthma trigger - damp
1786	Asthma trigger - animals
1787	Asthma trigger - seasonal
1788	Asthma trigger - cold air
1789	Asthma trigger - respiratory infection
2326	O/E - expiratory wheeze
6635	Increasing exercise wheeze
8793	Asthma control step 0
8794	Asthma control step 1
8795	Asthma control step 2
8796	Asthma control step 3
8797	Asthma control step 4
8798	Asthma control step 5
663e100	Asthma severely restricts exercise
173A.00	Exercise induced asthma
173B.00	Nocturnal cough / wheeze
173e.00	Viral wheeze
173e.11	Viral induced wheeze
178..00	Asthma trigger
178A.00	Asthma trigger - airborne dust
178B.00	Asthma trigger - exercise
1J70.00	Suspected asthma
1O2..00	Asthma confirmed
23D2.11	O/E - bronchospasm
663d.00	Emergency asthma admission since last appointment
663e.00	Asthma restricts exercise
663f.00	Asthma never restricts exercise
663J.00	Airways obstruction reversible
663j.00	Asthma - currently active
663K.00	Airways obstructn irreversible
663m.00	Asthma accident and emergency attendance since last visit
663N.00	Asthma disturbing sleep
663n.00	Asthma treatment compliance satisfactory
663N000	Asthma causing night waking
663N100	Asthma disturbs sleep weekly
663N200	Asthma disturbs sleep frequently
663O.00	Asthma not disturbing sleep
663O000	Asthma never disturbs sleep
663p.00	Asthma treatment compliance unsatisfactory
663P.00	Asthma limiting activities
663P000	Asthma limits activities 1 to 2 times per month

663P100	Asthma limits activities 1 to 2 times per week
663q.00	Asthma daytime symptoms
663Q.00	Asthma not limiting activities
663r.00	Asthma causes night symptoms 1 to 2 times per month
663s.00	Asthma never causes daytime symptoms
663t.00	Asthma causes daytime symptoms 1 to 2 times per month
663u.00	Asthma causes daytime symptoms 1 to 2 times per week
663v.00	Asthma causes daytime symptoms most days
663V.00	Asthma severity
663V000	Occasional asthma
663V100	Mild asthma
663V200	Moderate asthma
663V300	Severe asthma
663w.00	Asthma limits walking up hills or stairs
663x.00	Asthma limits walking on the flat
663y.00	Number of asthma exacerbations in past year
66Y9.00	Step up change in asthma management plan
66YC.00	Absent from work or school due to asthma
66YK.00	Asthma follow-up
66YP.00	Asthma night-time symptoms
66Yq.00	Asthma causes night time symptoms 1 to 2 times per week
66Yr.00	Asthma causes symptoms most nights
8H2P.00	Emergency admission, asthma
H060.11	Acute wheezy bronchitis
H30..12	Recurrent wheezy bronchitis
H302.00	Wheezy bronchitis
H312000	Chronic asthmatic bronchitis
H312011	Chronic wheezy bronchitis
H33..00	Asthma
H33..11	Bronchial asthma
H330.00	Extrinsic (atopic) asthma
H330.11	Allergic asthma
H330.12	Childhood asthma
H330.13	Hay fever with asthma
H330.14	Pollen asthma
H330000	Extrinsic asthma without status asthmaticus
H330011	Hay fever with asthma
H330100	Extrinsic asthma with status asthmaticus
H330111	Extrinsic asthma with asthma attack
H330z00	Extrinsic asthma NOS
H331.00	Intrinsic asthma
H331.11	Late onset asthma
H331000	Intrinsic asthma without status asthmaticus
H331100	Intrinsic asthma with status asthmaticus
H331111	Intrinsic asthma with asthma attack
H331z00	Intrinsic asthma NOS
H332.00	Mixed asthma
H333.00	Acute exacerbation of asthma
H334.00	Brittle asthma
H33z.00	Asthma unspecified
H33z.11	Hyperreactive airways disease

H33z000	Status asthmaticus NOS
H33z011	Severe asthma attack
H33z100	Asthma attack
H33z111	Asthma attack NOS
H33z200	Late-onset asthma
H33zz00	Asthma NOS
H33zz11	Exercise induced asthma
H33zz12	Allergic asthma NEC
H33zz13	Allergic bronchitis NEC
H35y700	Wood asthma
H47y000	Detergent asthma
H5y1600	Bronchospasm
R060900	[D]Wheezing
R060E00	[D]Mild wheeze
R060F00	[D]Moderate wheeze
R060G00	[D]Severe wheeze
R060H00	[D]Very severe wheeze

Appendix 2: READ codes for respiratory infections

Acute upper respiratory tract infections

1656	Feverish cold
7531100	Drainage of peritonsillar abscess
16L..00	Influenza-like symptoms
1C3..00	Earache symptoms
1C32.00	Unilateral earache
1C33.00	Bilateral earache
1C3Z.00	Earache symptom NOS
1C9..00	Sore throat symptom
1C9..11	Throat soreness
1C92.00	Has a sore throat
1C93.00	Persistent sore throat
1C9Z.00	Sore throat symptom NOS
1CB3.00	Throat pain
1CB3.11	Pain in throat
2D95.00	O/E - tympanic membrane red
2DB6.00	O/E - follicular tonsillitis
2DB7.00	O/E - exudate on tonsils
2DC1.00	O/E - pharynx hyperaemic
2DC2.00	O/E - granular pharyngitis
2DC3.00	Inflamed throat
4J3L.00	Influenza A virus H1N1 subtype detected
4JF4000	Throat swab culture positive
4JU0.00	Influenza H1 virus detected
4JU1.00	Influenza H2 virus detected
4JU2.00	Influenza H3 virus detected
4JU3.00	Influenza H5 virus detected
4JU4.00	Influenza A virus, other or untyped strain detected
4JU5.00	Influenza B virus detected
4JUE.00	Human rhinovirus detected
4JUF.00	Human parainfluenza virus detected
65VA.00	Notification of whooping cough
A32..00	Diphtheria
A320.00	Faucial diphtheria
A321.00	Nasopharyngeal diphtheria
A322.00	Anterior nasal diphtheria
A323.00	Laryngeal diphtheria
A32y.00	Other specified diphtheria
A32y000	Conjunctival diphtheria
A32yz00	Other specified diphtheria NOS
A32z.00	Diphtheria NOS
A33..00	Whooping cough
A33..11	Bordetella
A330.00	Bordetella pertussis
A331.00	Bordetella parapertussis
A33y.00	Whooping cough - other specified organism
A33yz00	Other whooping cough NOS
A33z.00	Whooping cough NOS
A34..00	Streptococcal sore throat and scarlatina

A340.00	Streptococcal sore throat
A340000	Streptococcal angina
A340100	Streptococcal laryngitis
A340200	Streptococcal pharyngitis
A340300	Streptococcal tonsillitis
A340z00	Streptococcal sore throat NOS
A34z.00	Streptococcal sore throat with scarlatina NOS
A552.00	Postmeasles otitis media
A793.00	Rhinovirus
Ayu3800	[X]Diphtheria, unspecified
Ayu3A00	[X]Whooping cough, unspecified
F51..00	Nonsuppurative otitis media + eustachian tube disorders
F510.00	Acute non suppurative otitis media
F510000	Acute otitis media with effusion
F510011	Acute secretory otitis media
F510100	Acute serous otitis media
F510200	Acute mucoid otitis media
F510300	Acute sanguinous otitis media
F510400	Acute allergic serous otitis media
F510600	Acute allergic sanguinous otitis media
F510z00	Acute nonsuppurative otitis media NOS
F514.00	Unspecified nonsuppurative otitis media
F514100	Serous otitis media NOS
F514200	Catarrhal otitis media NOS
F514300	Mucoid otitis media NOS
F514z00	Nonsuppurative otitis media NOS
F515.00	Eustachian tube salpingitis
F515.11	Catarrh - eustachian
F515000	Unspecified eustachian tube salpingitis
F515100	Acute eustachian tube salpingitis
F515z00	Eustachian tube salpingitis NOS
F52..00	Suppurative and unspecified otitis media
F520.00	Acute suppurative otitis media
F520000	Acute suppurative otitis media tympanic membrane intact
F520100	Acute suppurative otitis media tympanic membrane ruptured
F520300	Acute suppurative otitis media due to disease EC
F520z00	Acute suppurative otitis media NOS
F524.00	Purulent otitis media NOS
F524000	Bilateral suppurative otitis media
F525.00	Recurrent acute otitis media
F526.00	Acute left otitis media
F527.00	Acute right otitis media
F528.00	Acute bilateral otitis media
F52z.00	Otitis media NOS
F52z.11	Infection ear
F53..00	Mastoiditis and related conditions
F530.00	Acute mastoiditis
F530.11	Abscess of mastoid
F530.12	Empyema of mastoid
F530000	Acute mastoiditis without complications
F530100	Subperiosteal mastoid abscess

F530200	Gradenigo's syndrome
F530300	Acute mastoiditis with other complication
F530z00	Acute mastoiditis NOS
F53z.00	Mastoiditis NOS
F540.00	Acute myringitis without otitis media
F540100	Unspecified acute tympanitis
F540z00	Acute myringitis NOS
F587.00	Otalgia
F587000	Unspecified otalgia
F587100	Otogenic pain
F587z00	Otalgia NOS
FyuP000	[X]Other acute nonsuppurative otitis media
FyuP300	[X]Otitis media in bacterial diseases classified elsewhere
FyuP400	[X]Otitis media in viral diseases classified elsewhere
FyuP500	[X]Otitis media in other diseases classified elsewhere
FyuP700	[X]Other mastoiditis and related conditions
H00..00	Acute nasopharyngitis
H00..11	Common cold
H00..12	Coryza - acute
H00..13	Febrile cold
H00..14	Nasal catarrh - acute
H00..15	Pyrexial cold
H00..16	Rhinitis - acute
H01..00	Acute sinusitis
H01..11	Sinusitis
H010.00	Acute maxillary sinusitis
H010.11	Antritis - acute
H011.00	Acute frontal sinusitis
H012.00	Acute ethmoidal sinusitis
H013.00	Acute sphenoidal sinusitis
H014.00	Acute rhinosinusitis
H01y.00	Other acute sinusitis
H01y000	Acute pansinusitis
H01yz00	Other acute sinusitis NOS
H01z.00	Acute sinusitis NOS
H02..00	Acute pharyngitis
H02..11	Sore throat NOS
H02..12	Viral sore throat NOS
H02..13	Throat infection - pharyngitis
H020.00	Acute gangrenous pharyngitis
H021.00	Acute phlegmonous pharyngitis
H022.00	Acute ulcerative pharyngitis
H023.00	Acute bacterial pharyngitis
H023000	Acute pneumococcal pharyngitis
H023100	Acute staphylococcal pharyngitis
H023z00	Acute bacterial pharyngitis NOS
H024.00	Acute viral pharyngitis
H02z.00	Acute pharyngitis NOS
H03..00	Acute tonsillitis
H03..11	Throat infection - tonsillitis
H03..12	Tonsillitis

H030.00	Acute erythematous tonsillitis
H031.00	Acute follicular tonsillitis
H032.00	Acute ulcerative tonsillitis
H033.00	Acute catarrhal tonsillitis
H034.00	Acute gangrenous tonsillitis
H035.00	Acute bacterial tonsillitis
H035000	Acute pneumococcal tonsillitis
H035100	Acute staphylococcal tonsillitis
H035z00	Acute bacterial tonsillitis NOS
H036.00	Acute viral tonsillitis
H037.00	Recurrent acute tonsillitis
H03z.00	Acute tonsillitis NOS
H04..00	Acute laryngitis and tracheitis
H040.00	Acute laryngitis
H040000	Acute oedematous laryngitis
H040100	Acute ulcerative laryngitis
H040200	Acute catarrhal laryngitis
H040300	Acute phlegmonous laryngitis
H040400	Acute haemophilus influenzae laryngitis
H040600	Acute suppurative laryngitis
H040w00	Acute viral laryngitis unspecified
H040x00	Acute bacterial laryngitis unspecified
H040z00	Acute laryngitis NOS
H041.00	Acute tracheitis
H041000	Acute tracheitis without obstruction
H041100	Acute tracheitis with obstruction
H041z00	Acute tracheitis NOS
H042.00	Acute laryngotracheitis
H042.11	Laryngotracheitis
H042000	Acute laryngotracheitis without obstruction
H042100	Acute laryngotracheitis with obstruction
H042z00	Acute laryngotracheitis NOS
H043.00	Acute epiglottitis (non strep)
H043.11	Viral epiglottitis
H043000	Acute epiglottitis without obstruction
H043100	Acute epiglottitis with obstruction
H043200	Acute obstructive laryngitis
H043211	Croup
H043z00	Acute epiglottitis NOS
H044.00	Croup
H04z.00	Acute laryngitis and tracheitis NOS
H05..00	Other acute upper respiratory infections
H050.00	Acute laryngopharyngitis
H051.00	Acute upper respiratory tract infection
H052.00	Pharyngotracheitis
H053.00	Tracheopharyngitis
H054.00	Recurrent upper respiratory tract infection
H055.00	Pharyngolaryngitis
H05y.00	Other upper respiratory infections of multiple sites
H05z.00	Upper respiratory infection NOS
H05z.11	Upper respiratory tract infection NOS

H05z.12	Viral upper respiratory tract infection NOS
H120111	Catarrhal child
H121100	Atrophic pharyngitis
H121200	Granular pharyngitis
H121300	Hypertrophic pharyngitis
H130.12	Maxillary sinusitis
H131.11	Frontal sinusitis
H135.00	Recurrent sinusitis
H13y100	Pansinusitis
H141.00	Tonsil and/or adenoid hypertrophy
H15..00	Peritonsillar abscess - quinsy
H15..11	Quinsy
H1y1z14	Nasal infection
H1y2200	Parapharyngeal abscess
H1y2300	Retropharyngeal abscess
H1y2600	Pharynx or nasopharynx abscess
H27..00	Influenza
H271000	Influenza with laryngitis
H271100	Influenza with pharyngitis
H27z.00	Influenza NOS
H27z.11	Flu like illness
H27z.12	Influenza like illness
H29..00	Avian influenza
H2A..00	Influenza due to Influenza A virus subtype H1N1
H2A..11	Influenza A (H1N1) swine flu
Hyu0.00	[X]Acute upper respiratory infections
Hyu0000	[X]Other acute sinusitis
Hyu0100	[X]Acute pharyngitis due to other specified organisms
Hyu0200	[X]Acute tonsillitis due to other specified organisms
Hyu0400	[X]Flu+oth respiratory manifestations,'flu virus identified
Hyu0500	[X]Influenza+other manifestations,influenza virus identified
Hyu0600	[X]Influenza+oth respiratory manifestatns,virus not identifd
Hyu0700	[X]Influenza+other manifestations, virus not identified
R041.00	[D]Throat pain
R153200	[D]Positive culture findings in throat

Acute lower respiratory tract infections

1825	Pleuritic pain
1827	Painful breathing -pleurodynia
1827.11	Pleurodynia
23D5.00	O/E - pleural friction rub
23D5.11	O/E - pleural rub
4JU9.00	Respiratory syncytial virus A detected
4JUA.00	Respiratory syncytial virus B detected
4JUB.00	Respiratory syncytial virus untyped strain detected
4JUK.00	Mycoplasma pneumoniae detected
A022200	Salmonella pneumonia
A521.00	Varicella pneumonitis
A54x400	Herpes simplex pneumonia
A551.00	Postmeasles pneumonia

A730.00	Ornithosis with pneumonia
A741.00	Epidemic pleurodynia
A785000	Cytomegaloviral pneumonitis
A79A.00	Respiratory syncytial virus infection
A7y0200	Resp syncytial virus as cause of dis class to other chapters
AyuKN00	[X]Resp syncytial virus/cause/diseases classfd/oth chapters
H06..00	Acute bronchitis and bronchiolitis
H060.00	Acute bronchitis
H060.11	Acute wheezy bronchitis
H060000	Acute fibrinous bronchitis
H060100	Acute membranous bronchitis
H060200	Acute pseudomembranous bronchitis
H060300	Acute purulent bronchitis
H060400	Acute croupous bronchitis
H060500	Acute tracheobronchitis
H060600	Acute pneumococcal bronchitis
H060700	Acute streptococcal bronchitis
H060800	Acute haemophilus influenzae bronchitis
H060900	Acute neisseria catarrhalis bronchitis
H060A00	Acute bronchitis due to mycoplasma pneumoniae
H060B00	Acute bronchitis due to coxsackievirus
H060C00	Acute bronchitis due to parainfluenza virus
H060D00	Acute bronchitis due to respiratory syncytial virus
H060E00	Acute bronchitis due to rhinovirus
H060F00	Acute bronchitis due to echovirus
H060v00	Subacute bronchitis unspecified
H060w00	Acute viral bronchitis unspecified
H060x00	Acute bacterial bronchitis unspecified
H060z00	Acute bronchitis NOS
H061.00	Acute bronchiolitis
H061000	Acute capillary bronchiolitis
H061100	Acute obliterating bronchiolitis
H061200	Acute bronchiolitis with bronchospasm
H061300	Acute exudative bronchiolitis
H061400	Obliterating fibrous bronchiolitis
H061500	Acute bronchiolitis due to respiratory syncytial virus
H061600	Acute bronchiolitis due to other specified organisms
H061z00	Acute bronchiolitis NOS
H062.00	Acute lower respiratory tract infection
H06z.00	Acute bronchitis or bronchiolitis NOS
H06z112	Acute lower respiratory tract infection
H07..00	Chest cold
H2...00	Pneumonia and influenza
H20..00	Viral pneumonia
H20..11	Chest infection - viral pneumonia
H200.00	Pneumonia due to adenovirus
H201.00	Pneumonia due to respiratory syncytial virus
H202.00	Pneumonia due to parainfluenza virus
H20y.00	Viral pneumonia NEC
H20z.00	Viral pneumonia NOS
H21..00	Lobar (pneumococcal) pneumonia

H21..11	Chest infection - pneumococcal pneumonia
H22..00	Other bacterial pneumonia
H22..11	Chest infection - other bacterial pneumonia
H220.00	Pneumonia due to klebsiella pneumoniae
H221.00	Pneumonia due to pseudomonas
H222.00	Pneumonia due to haemophilus influenzae
H222.11	Pneumonia due to haemophilus influenzae
H223.00	Pneumonia due to streptococcus
H223000	Pneumonia due to streptococcus, group B
H224.00	Pneumonia due to staphylococcus
H22y.00	Pneumonia due to other specified bacteria
H22y000	Pneumonia due to escherichia coli
H22y011	E.coli pneumonia
H22y100	Pneumonia due to proteus
H22y200	Pneumonia - Legionella
H22yX00	Pneumonia due to other aerobic gram-negative bacteria
H22yz00	Pneumonia due to bacteria NOS
H22z.00	Bacterial pneumonia NOS
H23..00	Pneumonia due to other specified organisms
H23..11	Chest infection - pneumonia organism OS
H231.00	Pneumonia due to mycoplasma pneumoniae
H232.00	Pneumonia due to pleuropneumonia like organisms
H233.00	Chlamydial pneumonia
H23z.00	Pneumonia due to specified organism NOS
H24..00	Pneumonia with infectious diseases EC
H240.00	Pneumonia with measles
H241.00	Pneumonia with cytomegalic inclusion disease
H243.00	Pneumonia with whooping cough
H243.11	Pneumonia with pertussis
H24y.00	Pneumonia with other infectious diseases EC
H24y700	Pneumonia with varicella
H24yz00	Pneumonia with other infectious diseases EC NOS
H24z.00	Pneumonia with infectious diseases EC NOS
H25..00	Bronchopneumonia due to unspecified organism
H25..11	Chest infection - unspecified bronchopneumonia
H26..00	Pneumonia due to unspecified organism
H260.00	Lobar pneumonia due to unspecified organism
H261.00	Basal pneumonia due to unspecified organism
H263.00	Pneumonitis, unspecified
H270.00	Influenza with pneumonia
H270.11	Chest infection - influenza with pneumonia
H270000	Influenza with bronchopneumonia
H270100	Influenza with pneumonia, influenza virus identified
H270z00	Influenza with pneumonia NOS
H28..00	Atypical pneumonia
H2y..00	Other specified pneumonia or influenza
H2z..00	Pneumonia or influenza NOS
H30..00	Bronchitis unspecified
H30..11	Chest infection - unspecified bronchitis
H30..12	Recurrent wheezy bronchitis
H300.00	Tracheobronchitis NOS

H301.00	Laryngotracheobronchitis
H302.00	Wheezy bronchitis
H30z.00	Bronchitis NOS
H357.00	Pneumonitis
H501000	Pleural abscess
H501200	Pleural empyema
H501400	Purulent pleurisy
H51..00	Pleurisy
H510.00	Pleurisy without effusion or active tuberculosis
H510300	Acute dry pleurisy
H510400	Diaphragmatic pleurisy
H510500	Basal pleurisy
H510900	Pneumococcal pleurisy
H510A00	Staphylococcal pleurisy
H510B00	Streptococcal pleurisy
H510z00	Pleurisy without effusion or active tuberculosis NOS
H511.00	Bacterial pleurisy with effusion
H511000	Pneumococcal pleurisy with effusion
H511100	Staphylococcal pleurisy with effusion
H511z00	Bacterial pleurisy with effusion NOS
H51y.00	Other pleural effusion excluding mention of tuberculosis
H530300	Abscess of lung with pneumonia
H564.00	Bronchiolitis obliterans organising pneumonia
H56y100	Interstitial pneumonia
Hyu0800	[X]Other viral pneumonia
Hyu0A00	[X]Other bacterial pneumonia
Hyu0B00	[X]Pneumonia due to other specified infectious organisms
Hyu0D00	[X]Pneumonia in viral diseases classified elsewhere
Hyu0H00	[X]Other pneumonia, organism unspecified
Hyu1.00	[X]Other acute lower respiratory infections
Hyu1000	[X]Acute bronchitis due to other specified organisms
Hyu1100	[X]Acute bronchiolitis due to other specified organisms
R065400	[D]Pleuritic pain
R065500	[D]Pleurodynia

Part 2: codes for asthma medication

multilex	bnfcode	productname
0069001	03010200	ATROVENT aerosol inhaler 20micrograms/actuation [BOEH INGL]
0121001	03010103	BRICANYL aerosol inhaler [ASTRAZENEC]
0121002	03010103	BRICANYL TURBOHALER 500micrograms [ASTRAZENEC]
0121003	03010103	BRICANYL spacer inhaler [ASTRAZENEC]
0122001	03010103	BRICANYL unit dose nebuliser solution 5mg/2ml [ASTRAZENEC]
0122002	03010103	BRICANYL respirator solution 10mg/ml [ASTRAZENEC]
0281001	03010101<br>03010200<br>03010400	DUOVENT aerosol inhaler 40micrograms + 100micrograms/actuation [BOEH INGL]
0468001	03030100	INTAL SPINCAPS inhalation powder capsules [AVENTIS]
0602009	03010103	SALBUTAMOL aerosol inhaler 100micrograms/inhalation [BERK]
0603009	03010103	SALBUTAMOL aerosol inhaler 100micrograms/inhalation [CP PHARM]
0605009	03010103	SALBUTAMOL aerosol inhaler 100micrograms/inhalation [CELLTECH]
0606009	03010103	SALBUTAMOL aerosol inhaler 100micrograms/inhalation [HILLCROSS]
0743001	03010101<br>03010200<br>03010400	DUOVENT UDVs nebuliser solution [BOEH INGL]
0745001	03010101<br>03010200<br>03010400	ipratropium bromide with fenoterol hydrobromide unit dose nebulising solution 500micrograms + 1.25mg/4ml
0833001	12020100	RYNACROM capsules (for inhalation) [RPR/FISONS]
1020001	03010103	VENTOLIN aerosol inhaler 100micrograms/inhalation [GLAXO]
1020002	03010103	VENTOLIN ACCUHALER 200micrograms/actuation [GLAXO]
1020003	03010103	VENTOLIN EVOHALER 100micrograms/inhalation [GLAXO]
1021001	03010103	VENTOLIN ROTACAPS 200micrograms [A & H]
1021002	03010103	VENTOLIN ROTACAPS 400micrograms [A & H]
1023001	03010101	VENTOLIN respirator solution 5mg/ml [A & H]
1024001	03010101	VENTOLIN NEBULES unit dose nebulising solution 2.5mg [A & H]
1024002	03010101	VENTOLIN NEBULES unit dose nebulising solution 5mg [A & H]
1193003	03010103	SALBULIN aerosol inhaler [3M]
1195001	03010103	SALAMOL aerosol inhaler 100micrograms/actuation [IVAX]
1281001	03010102<br>03030100	INTAL COMPOUND capsules (for inhalation) [RHONE]
1340001	03010103	salbutamol capsules (for inhalation) 200micrograms
1340002	03010103	salbutamol capsules (for inhalation) 400micrograms
1340003	03010103	salbutamol CYCLOHALER
1419001	03010101<br>03020000	VENTIDE aerosol inhaler [A & H]
1561009	03010103	SALBUTAMOL aerosol inhaler 100micrograms/inhalation [IVAX]
1722001	03010200	ATROVENT UDVs nebuliser solution 0.25mg/ml [BOEH INGL]
1776001	03030100	INTAL aerosol inhaler [AVENTIS]
1776002	03030100	INTAL AUTOHALER 5mg/inhalation [AVENTIS]
1776003	03030100	INTAL SYNCRONER 5mg/inhalation [AVENTIS]
2032009	03010103	SALBUTAMOL CYCLOCAPS 200micrograms [DU PONT]
2032010	03010103	SALBUTAMOL CYCLOCAPS 400micrograms [DU PONT]
2049001	03030100	INTAL nebuliser solution 10mg/ml [AVENTIS]
2137001	03030100	STERI-NEB CROMOGEN nebuliser solution 10mg/ml [IVAX]
2177009	03010200	IPRATROPIUM BROMIDE inhalation solution 250micrograms/ml [GALEN]

2220001	03030100	CROMOGEN aerosol inhaler 5mg/inhalation [IVAX]
2221001	03010101	STERI-NEB SALAMOL unit dose nebulising solution 2.5mg/2.5ml [IVAX]
2221002	03010101	STERI-NEB SALAMOL unit dose nebulising solution 5mg/2.5ml [IVAX]
2319001	03020000	fluticasone unit dose nebulising suspension 2mg/2ml
2319002	03020000	fluticasone unit dose nebulising suspension 500micrograms/2ml
2327001	03020000	FLIXOTIDE NEBULES unit dose nebulising suspension 2mg/2ml [A & H]
2327002	03020000	FLIXOTIDE NEBULES unit dose nebulising suspension 500micrograms/2ml [A & H]
2414001	03010103	ASMAVEN aerosol inhaler 100micrograms [BERK]
2665001	03010200	ATROVENT FORTE aerosol inhaler 40micrograms/actuation [BOEH INGL]
2757001	03010103	BRICANYL refill canister [ASTRAZENEC]
2822009	03010103	TERBUTALINE unit dose nebulising solution 2.5mg/ml [GALEN]
2827001	03030100	TILADE aerosol inhaler 2mg/inhalation [SANOFI/AVE]
2827002	03030100	TILADE mint inhaler 2mg/inhalation [SANOFI/AVE]
2827003	03030100	TILADE mint SYNCRONER 2mg/inhalation [SANOFI/AVE]
2901009	03010101	SALBUTAMOL unit dose nebulising solution 2.5mg/2.5ml [GALEN]
2901010	03010101	SALBUTAMOL unit dose nebulising solution 5mg/2.5ml [GALEN]
2914001	03010103	salbutamol aerosol inhaler 100micrograms/inhalation
2914002	03010103	salbutamol cfc free inhaler 100micrograms/inhalation
3114009	03010200	IPRATROPIUM BROMIDE unit dose nebulising solution 250micrograms/ml [HILLCROSS]
3164009	03010103	SALBUTAMOL aerosol inhaler 100micrograms/inhalation [GEN (UK)]
3164011	03010101	SALBUTAMOL unit dose nebulising solution 5mg/2.5ml [GEN (UK)]
3263001	03030100	sodium cromoglicate capsules (for inhalation) 20mg
3264001	03010102<br>03030100	sodium cromoglicate with isoprenaline capsules (for inhalation)
3265001	03030100	sodium cromoglicate aerosol inhaler 1mg/inhalation
3265002	03030100	sodium cromoglicate inhaler and spacer 5mg/actuation
3265003	03030100	sodium cromoglicate nebuliser solution 10mg/ml
3266001	03030100	sodium cromoglicate aerosol inhaler 5mg/inhalation
3266002	03030100	sodium cromoglicate breath actuated inhaler 5mg/inhalation
3266003	03030100	sodium cromoglicate inhaler and spacer 5mg/inhalation
3268001	12020100	sodium cromoglicate capsules (for inhalation)
3371009	03010103	SALBUTAMOL cfc free inhaler 100micrograms/inhalation [APS]
3435009	03010103	SALBUTAMOL cfc free inhaler 100micrograms/inhalation [HILLCROSS]
3446001	03010101<br>03010200<br>03010400	fenoterol with ipratropium bromide aerosol inhaler 100micrograms + 40micrograms/actuation
3446002	03010101<br>03010200<br>03010400	fenoterol with ipratropium bromide breath actuated inhaler 100micrograms + 40micrograms/actuation
3574009	03010103	SALBUTAMOL cfc free inhaler 100micrograms/inhalation [KENT]
3834009	03010103	SALBUTAMOL aerosol inhaler 100micrograms/inhalation [KENT]
3886001	03010200	ipratropium bromide aerosol inhaler 20micrograms/dose
3886002	03010200	ipratropium bromide breath actuated inhaler 20micrograms/dose
3887001	03010200	ipratropium bromide aerosol inhaler 40micrograms/metered inhalation
3888001	03010200	ipratropium bromide nebuliser solution 0.25mg/ml
3888002	03010200	ipratropium bromide unit dose nebulising solution 250micrograms/ml
3888003	03010200	ipratropium bromide inhalation solution 250micrograms/ml
3889001	03010101<br>03010200<br>03010400	ipratropium bromide with fenoterol hydrobromide aerosol inhaler 40micrograms + 100micrograms/actuation

3889002	03010101<br>03010200<br>03010400	ipratropium bromide with fenoterol hydrobromide breath actuated inhaler 40micrograms + 100micrograms/actuation
3897009	03010103	SALBUTAMOL cfc free inhaler 100micrograms/inhalation [ACTAVIS]
3973009	03010103	SALBUTAMOL CYCLOCAPS capsules (for inhalation) 200micrograms [TEVA]
3974009	03010103	SALBUTAMOL CYCLOCAPS capsules (for inhalation) 400micrograms [TEVA]
4016001	03010101<br>03020000	beclometasone with salbutamol aerosol inhaler 50micrograms + 100micrograms/inhalation
4016002	03010101<br>03020000	beclometasone with salbutamol capsules (for inhalation) 200micrograms + 400micrograms
4016003	03010101<br>03020000	beclometasone with salbutamol capsules (for inhalation) 100micrograms + 200micrograms
4249001	03030100	nedocromil sodium aerosol inhaler 2mg/inhalation
4785001	03010103	terbutaline aerosol inhaler 250micrograms/actuation
4785002	03010103	terbutaline spacer inhaler 250micrograms/actuation
4785003	03010103	terbutaline refill canister 250micrograms/actuation
4786001	03010103	terbutaline unit dose nebuliser solution 5mg/2ml
4786002	03010103	terbutaline respirator solution 10mg/ml
5055001	03010101	salbutamol unit dose nebulising solution 2.5mg/2.5ml
5055002	03010101	salbutamol unit dose nebulising solution 5mg/2.5ml
5056001	03010103	salbutamol capsules (for inhalation) 200micrograms
5056002	03010103	salbutamol capsules (for inhalation) 400micrograms
5056003	03010101	salbutamol respirator solution 5mg/ml
5058001	03010101<br>03020000	salbutamol with beclometasone aerosol inhaler 100mcg + 50mcg
5058002	03010101<br>03020000	salbutamol with beclometasone capsules (for inhalation) 400micrograms + 200micrograms
5058003	03010101<br>03020000	salbutamol with beclometasone capsules (for inhalation) 200micrograms + 100micrograms
5137001	03010103	AEROLIN 400 aerosol inhaler 100micrograms/actuation [3M]
5138001	03010103	AEROLIN AUTOHALER breath actuated inhaler 100micrograms/actuation [3M]
5138002	03010103	AEROLIN AUTOHALER cfc free breath actuated inhaler 100micrograms/actuation [3M]
5150001	03010101<br>03020000	VENTIDE ROTACAPS [A & H]
5150002	03010101<br>03020000	VENTIDE paediatric ROTACAPS [A & H]
5153001	03010103	VENTODISKS disc 200micrograms/blister [A & H]
5153002	03010103	VENTODISKS disc 400micrograms/blister [A & H]
5374001	03020000<br>03010104	SERETIDE 250 EVOHALER cfc free inhaler [A & H]
5411009	03010200	IPRATROPIUM BROMIDE unit dose nebuliser solution 250micrograms/ml [GALEN]
5470001	03010103	SALBUVENT aerosol inhaler 100micrograms/actuation [PHARMACIA]
5544001	03010103	salbutamol disc 200micrograms
5544002	03010103	salbutamol disc 400micrograms
5616001	03010103	salbutamol breath actuated inhaler 100micrograms/actuation
5616002	03010103	salbutamol dry powder inhaler 200micrograms/actuation
5616003	03010103	salbutamol dry powder inhaler 95micrograms
5740001	03010101	SALBUVENT respirator solution 5mg/ml [PHARMACIA]
5800001	03010103	MAXIVENT aerosol inhaler 100micrograms/inhalation [ASHBOURNE]
6016001	03020000<br>03010104	fluticasone with salmeterol cfc free inhaler 50micrograms + 25micrograms/actuation

6016002	03020000<br>03010104	fluticasone with salmeterol cfc free inhaler 125micrograms + 25micrograms/actuation
6016003	03020000<br>03010104	fluticasone with salmeterol cfc free inhaler 250micrograms + 25micrograms/actuation
6267001	03010101<br>03010400<br>03030100	sodium cromoglicate with salbutamol aerosol inhaler
6267002	03010101<br>03010400<br>03030100	sodium cromoglicate with salbutamol inhaler and spacer
6268001	03010101<br>03010400<br>03030100	AEROCROM aerosol inhaler [CASTLEMEAD]
6268002	03010101<br>03010400<br>03030100	AEROCROM SYNCRONER [CASTLEMEAD]
6314001	03010200	ipratropium bromide capsules for inhalation + inhaler 40mcg
6314002	03010200	ipratropium bromide capsules (for inhalation) 40mcg
6315001	03010200	ATROVENT AEROHALER 40mcg [BOEH INGL]
6315002	03010200	ATROVENT AEROCAPS 40mcg [BOEH INGL]
6698009	03010101	SALBUTAMOL unit dose nebulising solution 2.5mg/2.5ml [HILLCROSS]
6817001	03010104	salmeterol aerosol inhaler 25micrograms/actuation
6817002	03010104	salmeterol disc 50micrograms
6817003	03010104	salmeterol dry powder inhaler 50micrograms/actuation
6818001	03010104	SEREVENT aerosol inhaler 25micrograms/actuation [GLAXO]
6818002	03010104	SEREVENT DISKHALER 50micrograms [GLAXO]
6818003	03010104	SEREVENT ACCUHALER 50micrograms/actuation [GLAXO]
6845001	03010103	terbutaline dry powder inhaler 500micrograms
6934001	03030100	INTAL FISONAIR aerosol inhaler 5mg/inhalation [AVENTIS]
6942001	03020000	FLIXOTIDE disc 50micrograms [A & H]
6942002	03020000	FLIXOTIDE disc 100micrograms [A & H]
6942003	03020000	FLIXOTIDE disc 250micrograms [A & H]
6943001	03020000	fluticasone disc 50micrograms
6943002	03020000	fluticasone disc 100micrograms
6943003	03020000	fluticasone disc 250micrograms
7099001	03020000	FLIXOTIDE aerosol inhaler 25micrograms/actuation [A & H]
7099002	03020000	FLIXOTIDE aerosol inhaler 50micrograms/actuation [A & H]
7099003	03020000	FLIXOTIDE aerosol inhaler 125micrograms/actuation [A & H]
7100001	03020000	fluticasone aerosol inhaler 25micrograms/actuation
7100002	03020000	fluticasone aerosol inhaler 50micrograms/actuation
7100003	03020000	fluticasone aerosol inhaler 125micrograms/actuation
7104001	03010200	ATROVENT AUTOHALER breath actuated inhaler 20micrograms/actuation [BOEH INGL]
7105001	03010101<br>03010200<br>03010400	DUOVENT AUTOHALER breath actuated inhaler [BOEH INGL]
7154001	03020000	fluticasone aerosol inhaler 250micrograms/actuation
7154002	03020000	fluticasone dry powder inhaler 50micrograms/inhalation
7154003	03020000	fluticasone dry powder inhaler 100micrograms/inhalation
7155001	03020000	fluticasone disc 500micrograms
7156001	03020000	FLIXOTIDE disc 500micrograms [A & H]

7157001	03020000	FLIXOTIDE aerosol inhaler 250micrograms/actuation [A & H]
7157002	03020000	FLIXOTIDE ACCUHALER 50micrograms/inhalation [A & H]
7157003	03020000	FLIXOTIDE ACCUHALER 100micrograms/inhalation [A & H]
7243001	03010101<br>03010200<br>03010400	COMBIVENT aerosol inhaler 20mcg + 100mcg [BOEH INGL]
7244001	03010101<br>03010200<br>03010400	ipratropium bromide with salbutamol aerosol inhaler 20mcg + 100mcg
7334001	03010200	STERI-NEB IPRATROPIUM unit dose nebulising solution 250micrograms/ml [IVAX]
7526001	03020000	fluticasone cfc free inhaler 125micrograms/actuation
7526002	03020000	fluticasone cfc free inhaler 250micrograms/actuation
7526003	03020000	fluticasone cfc free inhaler 50micrograms/actuation
7588001	03020000<br>03010104	budesonide with formoterol dry powder inhaler 400micrograms + 12micrograms/actuation
7800001	03020000<br>03010104	SERETIDE 500 ACCUHALER dry powder inhaler [GLAXO]
7949009	03010103	SALBUTAMOL cfc free inhaler 100micrograms/inhalation [NEOLAB]
8380001	03020000	FLIXOTIDE EVOHALER cfc free inhaler 125micrograms/actuation [A & H]
8380002	03020000	FLIXOTIDE EVOHALER cfc free inhaler 250micrograms/actuation [A & H]
8380003	03020000	FLIXOTIDE EVOHALER cfc free inhaler 50micrograms/actuation [A & H]
8429001	03010101	MAXIVENT STERIPOULE unit dose nebulising solution 2.5mg/2.5ml [ASHBOURNE]
8429002	03010101	MAXIVENT STERIPOULE unit dose nebulising solution 5mg/2.5ml [ASHBOURNE]
8452001	03020000<br>03010104	SERETIDE 125 EVOHALER cfc free inhaler [A & H]
8506001	03020000<br>03010104	SYMBICORT TURBOHALER dry powder inhaler 400micrograms + 12micrograms/actuation [ASTRAZENEC]
8602001	03010103	AIROMIR cfc free inhaler 100micrograms/inhalation [TEVA]
8651001	03020000<br>03010104	SERETIDE 250 ACCUHALER dry powder inhaler [GLAXO]
8654001	03010103	SALAMOL cfc free inhaler 100micrograms/inhalation [KENT]
8664001	03010103	AIROMIR AUTOHALER cfc free breath actuated inhaler 100micrograms/actuation [IVAX]
8665001	03020000	FLIXOTIDE ACCUHALER 250micrograms/inhalation [A & H]
8665002	03020000	FLIXOTIDE ACCUHALER 500micrograms/inhalation [A & H]
8677001	03020000	fluticasone dry powder inhaler 250micrograms/inhalation
8677002	03020000	fluticasone dry powder inhaler 500micrograms/inhalation
8679001	03010101	SALAMOL STERI-NEB unit dose nebulising solution 2.5mg/2.5ml [NUMARK]
8688001	03010101<br>03010200<br>03010400	COMBIVENT UDVs nebuliser solution 2.5ml [BOEH INGL]
8689001	03010101<br>03010200<br>03010400	ipratropium bromide with salbutamol unit dose nebulising solution 500micrograms + 2.5mg/2.5ml
8715001	03010103	SALAMOL EASI-BREATHE breath actuated inhaler 100micrograms/actuation [IVAX]
8715002	03010103	SALAMOL EASI-BREATHE cfc free breath actuated inhaler 100micrograms/actuation [IVAX]
8727001	03010103	salbutamol breath actuated inhaler 100micrograms/actuation
8892001	03010101<br>03010200<br>03010400	salbutamol with ipratropium bromide unit dose nebulising solution 2.5mg + 500micrograms/2.5ml

8921001	03010101<br>03010200<br>03010400	fenoterol with ipratropium bromide unit dose nebulising solution 1.25mg + 500micrograms/4ml
9056001	03010104	FORADIL capsules (for inhalation) 12mcg [NOV/CIBA]
9057001	03010104	formoterol fumarate capsules (for inhalation) 12mcg
9319001	03010101<br>03010200<br>03010400	salbutamol with ipratropium bromide aerosol inhaler 100micrograms + 20micrograms/actuation
9412001	03010103	SALAMOL cfc free inhaler 100micrograms/inhalation [IVAX]
9580001	03010103	salbutamol vortex metered dose inhaler 100micrograms/inhalation
9581001	03010103	SALBUTAMOL SPACEHALER 100micrograms/inhalation [CELLTECH]
9605001	03020000<br>03010104	SYMBICORT TURBOHALER dry powder inhaler 100micrograms + 6micrograms/actuation [ASTRAZENEC]
9605002	03020000<br>03010104	SYMBICORT TURBOHALER dry powder inhaler 200micrograms + 6micrograms/actuation [ASTRAZENEC]
10652001	03010103	PULVINAL SALBUTAMOL dry powder inhaler 200micrograms/actuation [CHIESI]
10713001	03030100	CROMOGEN EASI-BREATHE breath actuated inhaler 5mg/inhalation [IVAX]
10724001	03010103	SPACEHALER SALBUTAMOL SPACEHALER 100micrograms/inhalation [CELLTECH]
10821001	03010101	SALAMOL STERI-NEB unit dose nebulising solution 5mg/2.5ml [NUMARK]
10939001	03010103	SALBULIN cfc free inhaler 100micrograms/actuation [3M]
11165001	03010103	VENTOLIN EASI-BREATHE breath actuated inhaler 100micrograms/actuation [A & H]
11474001	03020000<br>03010104	salmeterol with fluticasone dry powder inhaler 50micrograms + 100micrograms/inhalation
11474002	03020000<br>03010104	salmeterol with fluticasone dry powder inhaler 50micrograms + 250micrograms/inhalation
11474003	03020000<br>03010104	salmeterol with fluticasone dry powder inhaler 50micrograms+ 500micrograms/inhalation
11475001	03020000<br>03010104	fluticasone with salmeterol dry powder inhaler 100micrograms + 50micrograms/inhalation
11475002	03020000<br>03010104	fluticasone with salmeterol dry powder inhaler 250micrograms + 50micrograms/inhalation
11475003	03020000<br>03010104	fluticasone with salmeterol dry powder inhaler 500micrograms + 50micrograms/inhalation
11477001	03020000<br>03010104	SERETIDE 100 ACCUHALER dry powder inhaler [GLAXO]
11509001	03010104	formoterol fumarate dry powder inhaler 6 micrograms/actuation
11509002	03010104	formoterol fumarate dry powder inhaler 12micrograms/actuation
11511001	03010104	OXIS 6 TURBOHALER dry powder inhaler 6 micrograms/actuation [ASTRAZENEC]
11512001	03010104	OXIS 12 TURBOHALER dry powder inhaler 12micrograms/actuation [ASTRAZENEC]
11529001	03010103	ASMASAL SPACEHALER 100micrograms/inhalation [CELLTECH]
11642001	03010103	ASMASAL CLICKHALER dry powder inhaler 95micrograms [FOCUS]
11647001	03030200	SINGULAIR PAEDIATRIC chewable tablet 5mg [M S D]
11647002	03030200	SINGULAIR PAEDIATRIC chewable tablet 4mg [M S D]
11648001	03030200	montelukast (as sodium salt) chewable tablet 5mg
11648002	03030200	montelukast (as sodium salt) tablets 10mg
11648003	03030200	montelukast (as sodium salt) chewable tablet 4mg
11657001	03030200	SINGULAIR tablets 10mg [M S D]
11694001	03020000<br>03010104	budesonide with formoterol dry powder inhaler 100micrograms + 6micrograms/actuation
11694002	03020000	budesonide with formoterol dry powder inhaler 200micrograms +

	03010104	6micrograms/actuation
11700001	03010200	RESPONTIN NEBULES unit dose nebulising solution 250micrograms/ml [GLAXO]
11726001	03010200	TROPIOVENT STERIPOULE unit dose nebulising solution 250micrograms/ml [ASHBOURNE]
11737001	03010103	SALAMOL cfc free inhaler 100micrograms/inhalation [SANDOZ]
11897001	03030200	zafirlukast tablets 20mg
11904001	03030200	ACCOLATE tablets 20mg [ASTRAZENEC]
11995001	03010103	salbutamol cfc free breath actuated inhaler 100micrograms/actuation
12019001	03020000<br>03010104	salmeterol with fluticasone cfc free inhaler 25micrograms + 50micrograms/actuation
12019002	03020000<br>03010104	salmeterol with fluticasone cfc free inhaler 25micrograms + 125micrograms/actuation
12019003	03020000<br>03010104	salmeterol with fluticasone cfc free inhaler 25micrograms + 250micrograms/actuation
12020001	03020000<br>03010104	SERETIDE 50 EVOHALER cfc free inhaler [A & H]
12398001	03030200	montelukast (as sodium salt) granules 4mg/sachet
12402001	03030200	SINGULAIR PAEDIATRIC granules 4mg/sachet [M S D]
12485001	03010200	ipratropium bromide cfc free inhaler 20micrograms/actuation
12486001	03010200	ATROVENT cfc free inhaler 20micrograms/actuation [BOEH INGL]
12722001	03010103	salbutamol dry powder inhaler 100micrograms/actuation
12723001	03010103	EASYHALER SALBUTAMOL dry powder inhaler 100micrograms/actuation [ORION]
12724001	03010103	EASYHALER SALBUTAMOL dry powder inhaler 200micrograms/actuation [ORION]
12871001	03010200	ipratropium bromide unit dose nebuliser solution 250micrograms/ml
12872001	03010200	ipratropium bromide unit dose nebuliser solution 500micrograms/2ml
12873001	03010200	ATROVENT UDVs nebuliser solution 250micrograms/1ml [BOEH INGL]
12874001	03010200	ATROVENT UDVs nebuliser solution 500micrograms/2ml [BOEH INGL]
12890001	03010200	RESPONTIN NEBULES unit dose nebulising solution 250micrograms/ml [GLAXO]
12891001	03010200	RESPONTIN NEBULES unit dose nebulising solution 500micrograms/2ml [GLAXO]
12892001	03010200	STERI-NEB IPRATROPIUM unit dose nebulising solution 250micrograms/ml [IVAX]
12894001	03010200	STERI-NEB IPRATROPIUM unit dose nebulising solution 500micrograms/2ml [IVAX]
13469001	03010104	formoterol fumarate cfc free inhaler 12micrograms/actuation
13470001	03010104	ATIMOS MODULITE cfc free inhaler 12micrograms/actuation [CHIESI]
13502001	03030100	nedocromil sodium cfc free inhaler 2mg/inhalation
13503001	03030100	TILADE cfc free inhaler 2mg/inhalation [SANOFI/AVE]
13678001	03010104	salmeterol cfc free inhaler 25micrograms/actuation
13679001	03010104	SEREVENT EVOHALER cfc free inhaler 25micrograms/actuation [GLAXO]
14551001	03010101<br>03010200<br>03010400	IPRAMOL STERI-NEB unit dose nebulising solution 500micrograms + 2.5mg/2.5ml [IVAX]
15010001	03010104	EASYHALER FORMOTEROL dry powder inhaler 12micrograms/actuation [ORION]
15084001	03010104	salmeterol inhalation powder blisters with device 50micrograms
15087001	03010104	salmeterol inhalation powder blisters (refill) 50micrograms
15088001	03010104	SEREVENT DISKHALER inhalation powder 50micrograms [GLAXO]
15091001	03010104	SEREVENT DISKHALER (REFILL) inhalation powder 50micrograms [GLAXO]
15228001	03020000	fluticasone inhalation powder blisters with device 50micrograms
15229001	03020000	fluticasone inhalation powder blisters (refill) 50micrograms
15231001	03020000	FLIXOTIDE DISKHALER inhalation powder 50micrograms [A & H]
15232001	03020000	FLIXOTIDE DISKHALER (REFILL) inhalation powder 50micrograms [A & H]
15236001	03020000	fluticasone inhalation powder blisters with device 100micrograms

15237001	03020000	fluticasone inhalation powder blisters (refill) 100micrograms
15239001	03020000	FLIXOTIDE DISKHALER inhalation powder 100micrograms [A & H]
15240001	03020000	FLIXOTIDE DISKHALER (REFILL) inhalation powder 100micrograms [A & H]
15242001	03020000	fluticasone inhalation powder blisters with device 250micrograms
15243001	03020000	fluticasone inhalation powder blisters (refill) 250micrograms
15244001	03020000	FLIXOTIDE DISKHALER inhalation powder 250micrograms [A & H]
15245001	03020000	FLIXOTIDE DISKHALER (REFILL) inhalation powder 250micrograms [A & H]
15246001	03020000	fluticasone inhalation powder blisters with device 500 micrograms
15247001	03020000	fluticasone inhalation powder blisters (refill) 500 micrograms
15248001	03020000	FLIXOTIDE DISKHALER inhalation powder 500 micrograms [A & H]
15249001	03020000	FLIXOTIDE DISKHALER (REFILL) inhalation powder 500 micrograms [A & H]
15760001	03020000<br>03010104	beclometasone extrafine particle with formoterol cfc free inhaler 100micrograms + 6micrograms/actuation
15761001	03020000<br>03010104	FOSTAIR cfc free inhaler 100micrograms + 6micrograms/actuation [CHIESI]
16041001	03010103	salbutamol dry powder inhalation cartridge with device 100micrograms
16042001	03010103	salbutamol dry powder inhalation cartridge (refill) 100micrograms
16043001	03010103	SALBULIN MDPI NOVOLIZER dry powder inhalation cartridge with device 100micrograms [MEDA]
16044001	03010103	SALBULIN MDPI NOVOLIZER dry powder inhalation cartridge (refill) 100micrograms [MEDA]
16313001	03030100	sodium cromoglicate cfc free inhaler 5mg
16320001	03030100	INTAL cfc free inhaler 5mg [SANOFI/AVE]
16659001	03010200	STERIPOULE IPRATROPIUM unit dose nebuliser solution 250micrograms/ml [GALEN]
16663001	03010200	STERIPOULE IPRATROPIUM unit dose nebuliser solution 500micrograms/2ml [GALEN]
16667001	03010101	STERIPOULE SALBUTAMOL unit dose nebuliser solution 2.5mg/2.5ml [GALEN]
16669001	03010101	STERIPOULE SALBUTAMOL unit dose nebuliser solution 5mg/2.5ml [GALEN]
17182001	03010101<br>03010200<br>03010400	SALIPRANEB unit dose nebulising solution 500micrograms + 2.5mg/2.5ml [BREATH]
17736001	03010200	ATROVENT AEROHALER inhalation powder capsules with device 40mcg [BOEH INGL]
17737001	03010200	ATROVENT AEROCAPS inhalation powder capsules 40mcg [BOEH INGL]
17738001	03010103	BRICANYL TURBOHALER dry powder inhaler 500micrograms [ASTRAZENEC]
17739001	03010103	VENTOLIN EVOHALER cfc free inhaler 100micrograms/inhalation [GLAXO]
17740001	03010103	VENTOLIN ACCUHALER dry powder inhaler 200micrograms/actuation [GLAXO]
17741001	03020000	FLIXOTIDE ACCUHALER dry powder inhaler 50micrograms/inhalation [A & H]
17742001	03020000	FLIXOTIDE ACCUHALER dry powder inhaler 100micrograms/inhalation [A & H]
17744001	03020000	FLIXOTIDE ACCUHALER dry powder inhaler 250micrograms/inhalation [A & H]
17745001	03020000	FLIXOTIDE ACCUHALER dry powder inhaler 500micrograms/inhalation [A & H]
17804001	03010103	BRICANYL RESPULES unit dose nebuliser solution 5mg/2ml [ASTRAZENEC]
